# Impact of Dendrimer Terminal Group Chemistry on Blockage of the Anthrax Toxin Channel: A Single Molecule Study

**DOI:** 10.3390/toxins8110337

**Published:** 2016-11-15

**Authors:** Goli Yamini, Nnanya Kalu, Ekaterina M. Nestorovich

**Affiliations:** Department of Biology, The Catholic University of America, Washington DC, WA 20064, USA; 00yamini@cua.edu (G.Y.); 56kalu@cua.edu (N.K.)

**Keywords:** multivalency, planar lipid bilayer technique, *Bacillus anthracis*, protective antigen, pore blockage

## Abstract

Nearly all the cationic molecules tested so far have been shown to reversibly block K^+^ current through the cation-selective PA_63_ channels of anthrax toxin in a wide nM–mM range of effective concentrations. A significant increase in channel-blocking activity of the cationic compounds was achieved when multiple copies of positively charged ligands were covalently linked to multivalent scaffolds, such as cyclodextrins and dendrimers. Even though multivalent binding can be strong when the individual bonds are relatively weak, for drug discovery purposes we often strive to design multivalent compounds with high individual functional group affinity toward the respective binding site on a multivalent target. Keeping this requirement in mind, here we perform a single-channel/single-molecule study to investigate kinetic parameters of anthrax toxin PA_63_ channel blockage by second-generation (G2) poly(amido amine) (PAMAM) dendrimers functionalized with different surface ligands, including G2-NH_2_, G2-OH, G2-succinamate, and G2-COONa. We found that the previously reported difference in *IC*_50_ values of the G2-OH/PA_63_ and G2-NH_2_/PA_63_ binding was determined by both on- and off-rates of the reversible dendrimer/channel binding reaction. In 1 M KCl, we observed a decrease of about three folds in $k_{on}$ and a decrease of only about ten times in $t_{res}$ with G2-OH compared to G2-NH_2_. At the same time for both blockers, $k_{on}$ and $t_{res}$ increased dramatically with transmembrane voltage increase. PAMAM dendrimers functionalized with negatively charged succinamate, but not carboxyl surface groups, still had some residual activity in inhibiting the anthrax toxin channels. At 100 mV, the on-rate of the G2-succinamate binding was comparable with that of G2-OH but showed weaker voltage dependence when compared to G2-OH and G2-NH_2_. The residence time of G2-succinamate in the channel exhibited opposite voltage dependence compared to G2-OH and G2-NH_2_, increasing with the *cis*-negative voltage increase. We also describe kinetics of the PA_63_ ion current modulation by two different types of the “imperfect” PAMAM dendrimers, the mixed-surface G2 75% OH 25% NH_2_ dendrimer and G3-NH_2_ dendron. At low voltages, both “imperfect” dendrimers show similar rate constants but significantly weaker voltage sensitivity when compared with the intact G2-NH_2_ PAMAM dendrimer.

## 1. Introduction

**Figure 1:**
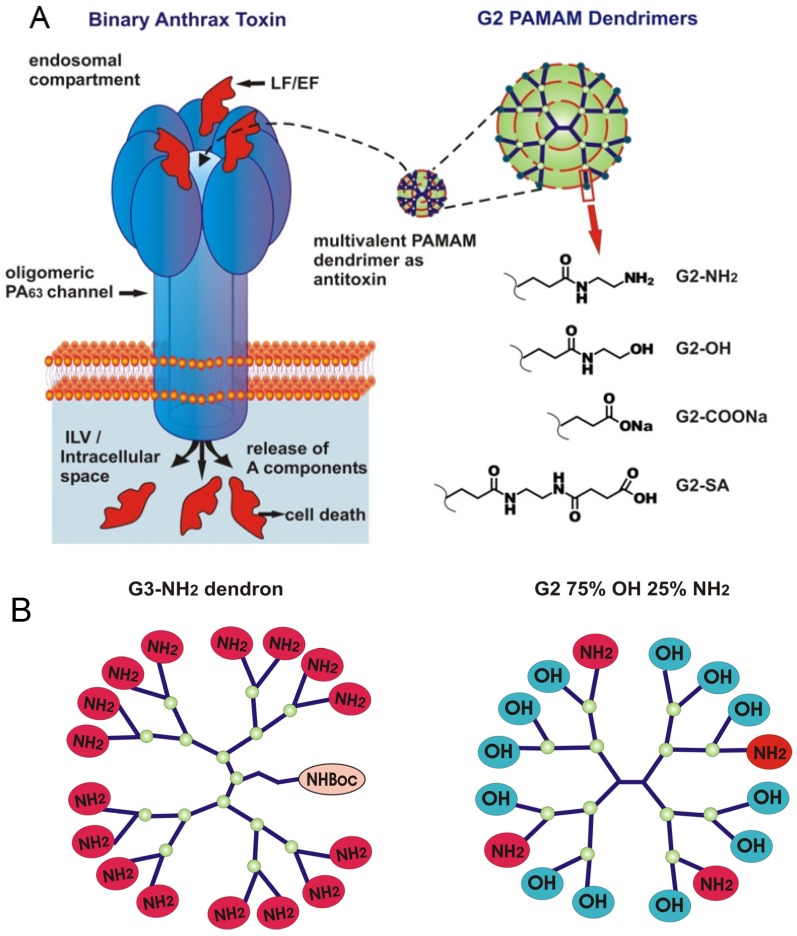
Inhibition of anthrax toxin by poly(amido amine) (PAMAM) dendrimers. (**A**) Oligomeric PA_63_ channel, produced by *B*. *anthracis* (left), is responsible for translocation of lethal factor (LF) and edema factor (EF) into the host cell. The cartoon is a simplified illustration of the early/late endosomal stages of the LF and EF host cell transport. Second generation (G2) PAMAM dendrimers (right) tested in this study. All shown G2 dendrimers have 16 terminal groups that could be charged positively (G2-NH_2_), negatively (G2-COONa, G2-SA) or are neutral (G2-OH); (**B**) Schematic representation of the mixed-surface G3-NH_2_ dendron (left) and G2 75% OH 25% NH_2_ dendrimer (right). Note that in contrast to all other dendrimers, G2 75% OH 25% NH_2_ is not monodisperse and contains 75% of terminal OH groups and 25% of terminal NH_2_ groups on average.

Many bacterial exotoxins oligomerize during invasion to form ion-conductive channels or pores in the host cell or organelle membranes. This oligomeric centrosymmetric organization represents an ideal multivalent receptor target to explore a variety of multivalent channel-blocking ligands with a controlled number of preassembled functional groups (reviewed in [1]). In the past decade, two classes of multivalent compounds, cationic cyclodextrins (CDs) and dendrimers have been reported to directly block the channel-forming B components of the AB type anthrax, C2, iota, and CDT toxins [2,3,4,5], PA_63_, C2IIa, Ib, and CDTb, respectively. The potency of these multivalent blockers compares well with the most effective blockers of the classical channels of electrophysiology (Table 3 in ref. [6]) and exceeds activities of the small-molecule cationic ligands [7,8,9,10,11]. A key advantage of cyclodextrins (reviewed in [12]) is their arrangement into rigid 6-, 7-, and 8-fold centrosymmetric structures with controlled number and position of potential attachment sites and the ability to form water-soluble “host-guest” inclusion complexes with poorly soluble small molecules and macromolecule fragments. The poly(amido amine) (PAMAM) dendrimers (reviewed in [13]) are repeatedly branched polymers with all bonds forming amidoamine branches emanating from a central alkyldiamine core, where each consecutive growth step represents a new dendrimer “generation” with an increased diameter and doubled number of reactive surface functional groups. PAMAM dendrimers form monodispersed, starburst-shaped polymers that are synthesized in generations with a growing but well-controlled number of attachment sites (Figure S1). While many details of the biophysical mechanisms of cyclodextrin interaction with the channel-forming components of anthrax, C2, and iota AB type toxins are known [14,15], the physical forces involved in the dendrimer/channel binding reaction require further analysis. In this small-scale study written for the special issue of Toxins (Basel) on “Novel Pharmacological Inhibitors for Bacterial Protein Toxins”, we perform a single channel investigation of the PAMAM dendrimer/PA_63_ channel binding reaction focusing on two specific aspects of the dendrimer-induced channel blockage. Firstly, we perform a single-channel analysis of the kinetic parameters of the dendrimer/PA_63_ binding reaction using generation 2 amino-terminated (G2-NH_2_), hydroxyl-terminated (G2-OH), succinamate-terminated (G2-SA), and carboxyl-terminated (G2-COONa) PAMAM dendrimers (Figure 1A and Figure S1). The amino-terminated PAMAM dendrimers of different generations were recently reported to effectively block the PA_63_ channel lumen in multichannel experiments under near-physiological conditions [5]. This effect was explained by the direct electrostatic interaction of the positively-charged terminal amino-groups on the PAMAM dendrimers with the negatively charged lumen of PA_63_. However, second and third generation (G2 and G3) PAMAM dendrimers functionalized with surface hydroxyl groups were reported to have an avidity decrease of only 20 and 9 times compared to the G2 and G3 amino-terminated dendrimers, respectively. This difference is comparable with the variation observed between different PAMAM dendrimer generations. Moreover, even PAMAM dendrimers terminated with negatively charged carboxylic and succinamic surface groups showed some residual PA_63_ binding. When fine-tuned, some of these dendrimers may offer the advantage of being effective channel inhibitors with decreased cytotoxicity. In fact, while all dendrimers are less toxic than linear polymers [16,17], the cationic amino-terminated PAMAM dendrimers have been reported to display concentration- and generation-dependent cytotoxicity, and are therefore less biocompatible compared to their neutral and negatively charged analogues [18,19]. Secondly, we analyze the kinetic parameters of the blockage of a single PA_63_ channel by G2 75% OH 25% NH_2_ PAMAM dendrimer and G3-NH_2_ dendron (Figure 1B). It has been demonstrated that a favorable therapeutic window for dendrimers can also be achieved by either partial surface modification, aiming to lower amino group density, or by degradation of the dendrimers to “imperfect” dendrimers or fractured dendron-like branches [20]. The channel-blocking activity of these two types of the “imperfect” dendrimers was previously investigated on a multichannel level [5]. The mixed-surface G2 75% OH 25% NH_2_ PAMAM dendrimer that, on average, had only four surface positive charges was about 17 times less active (*IC*_50_ = 122 nM vs. 7 nM) than the 16+ charged G2-NH_2_ dendrimer, and its activity was comparable (*IC*_50_ = 122 nM vs. 128 nM) with the 4+ charged G0 dendrimer. The structurally incomplete 4+ charged G1 dendron was about 26 times more effective (*IC*_50_ = 4.9 nM) than the 4+ charged G0 dendrimer (*IC*_50_ = 128 nM). The 8+ charged G2 dendron and G1 dendrimer had similar inhibitory activity. The five different commercially available generation 2 dendrimers and one generation 3 dendron, investigated in this study, were chosen with the purpose of specifically focusing on blocker terminal group chemistry and dendrimer flexibility. The number of terminal amino, hydroxyl, succinamate, carboxyl, or mixed OH/NH_2_ surface groups was fixed and equal to 16.

In this study, we investigated the oligomeric channel-forming B component of the anthrax toxin, protective antigen (PA), as a multivalent target for the multivalent dendrimer binding. Traditionally, PA, because of its main role in anthrax toxin uptake, has been one of the key targets for small molecule and multivalent antitoxin development [21]. The AB type anthrax toxin is composed of three individually nontoxic proteins. The two A components, lethal factor (LF) and edema factor (EF), are the intracellularly active enzymes. LF is a Zn-metalloprotease that cleaves MAP kinase kinases [22,23] and Nlrp1 [24]. EF is a Ca^2+^ and calmodulin-activated adenylyl cyclase [25,26]. Protective antigen (PA), named this way for its ability to elicit protective antibodies (the property utilized in the anthrax vaccines), is a receptor for LF/EF binding, which mediates their translocation. The anthrax toxin intracellular delivery occurs in several stages. After binding to its cellular CMG2 and/or TEM8 receptors and proteolytic cleavage to PA_63_, by extracellular furin, PA oligomerizes on the host cell surface to form heptameric [27] and/or oligomeric ring-shaped pre-pores [28,29] creating three [30] or four [28] LF and/or EF binding sites. After receptor-mediated endocytosis [31], the anthrax toxin AB complexes are delivered to the acidic environment of the early endosome. There, the PA oligomers undergo substantial conformational changes leading to their insertion in endosomal limiting, and possibly in intraluminal vesicle membranes [32], eventually forming an extended 180-Å long “flower-on-a-stem” cation-selective [33] channel with a 75 Å long bud and a 105 Å long stem and radius varying from 16 Å to 3.5 Å [34]. This channel is generally believed to work as an effective translocase that unfolds and allows for translocation of LF and EF into the cytosol under a pH gradient across the late endosomal limiting membrane (pH_endosome_ < pH_cytosol_) [35,36]. An alternative model suggests that the anthrax toxin catalyzes the rupture of the endosomal membranes, which leads to the consequent delivery of the toxin complexes into the cytosol [37].

## 2. Results

### 2.1. Two Modes of G2-NH_2_ PAMAM Dendrimer Inhibition of PA_63_ Channel

**Figure 2:**
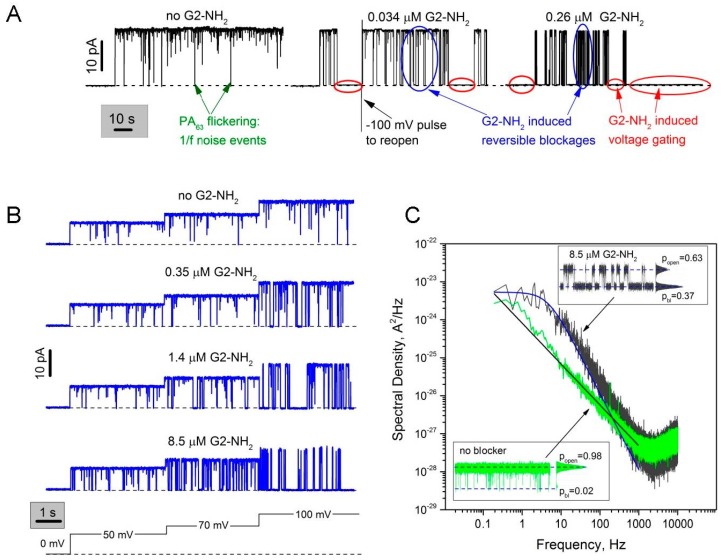
Modulation of a single PA_63_ channel current by G2-NH_2_ PAMAM dendrimer. (**A**) Two modes of G2-NH_2_ action on a single PA_63_ channel. In the absence of G2-NH_2_ (left), PA_63_ remains in an open state. Fast flickering between the open and closed states (the so-called 1/*f* noise) is mostly but not completely removed by averaging over a time interval of 50 ms. In the presence of two different G2-NH_2_ concentrations (middle and right), both blocker-induced reversible blockage and prolonged voltage gating events are seen. Recordings were taken at 100 mV applied voltage; (**B**) First mode of action. In the absence of the blocker (top) the ion movement is determined by the geometry and surface properties of the PA_63_ pore. In the presence of G2-NH_2_ in the *cis* compartment of the bilayer chamber (three following rows), the channel gets reversibly blocked. At higher concentrations of G2-NH_2_ (bottom) the blockages, which are seen as downward spikes, are more frequent. The probability of finding PA_63_ in the blocked state increases with *cis*-positive transmembrane voltage increase (50, 70, and 100 mV are shown). Current tracks were averaged over a time interval of 2 ms; (**C**) Power spectral densities of the G2-NH_2_–induced PA_63_ current fluctuations (spectrum in grey) can be fitted by a single Lorentzian at frequencies of <1000 Hz in contrast to 1/*f* noise in the absence of G2-NH_2_ (spectrum in green). A clear deviation from a single Lorentzian dependence at *f* > 100 Hz could to some degree be explained by the 1/*f* noise and the partial dendrimer degradation and breakage with formation of the imperfect cationic substrates, capable of blocking PA_63_ channels with shorter lifetimes (mass spectra and NMR characterization is given in Figures S5 and S6). Inserts: 1-s current tracks averaged over a time interval of 0.2 ms for blocker-free (bottom left) and 8.5 μM G2-NH_2_ (upper right) recordings. All-point histograms are shown to the right of the recordings; $p_{open}$ and $p_{bl}$ denote the probability of the PA_63_ channel being in conductive and non-conductive states, respectively. Applied voltage was 100 mV; dashed lines represent zero current levels.

Figure 2 illustrates the bimodal effect of G2-NH_2_ PAMAM *cis*-solution addition on the ionic current through a single PA_63_ channel incorporated into planar lipid bilayer membranes. To obtain reliable statistics on G2-NH_2_/PA_63_ interaction, we performed the single channel measurements in 1 M KCl. A decrease in salt concentration led to a dramatic increase in the blocker lifetimes, suggesting the involvement of the long-range Coulomb interactions. Quantitative analysis of the process at lower, e.g., physiological salt concentrations, proved impossible over the course of our experiments. Previously, the PAMAM dendrimers were reported to be ~100–900 times more effective when added to the *cis*-side of the membrane, which is also the side of PA_63_ addition [5]. PA_63_ insertion was shown to be almost exclusively unidirectional [2,9,33], with the bud, LF/EF binding part of the channel, facing the *cis*-side solution (corresponding to the endosome interior), and the stem part facing the *trans*-side solution (corresponding to the cytosol or ILV interior). The single channel current recordings show that, in a manner similar to the cationic β-cyclodextrin [14] and G1-NH_2_ PAMAM dendrimer blockers [5], the G2-NH_2_ inhibitive action is bimodal (Figure 2A). Firstly, G2-NH_2_ addition generates complete but reversible blockages of ion current through a single channel (marked with two blue ovals, Figure 2A). Frequency of these events increases in a concentration-dependent manner and is a strong function of the applied transmembrane voltage (Figure 2B). Note: *cis*-positive sign of the applied transmembrane voltages corresponds to the inside-positive voltage gradient across endosomal limiting membranes. Secondly, G2-NH_2_ addition led to a dramatic increase in the voltage-dependent gating of PA_63_ channels (Figure S2), seen as prolonged closing events (marked with red ovals, Figure 2A, middle and right). Higher concentrations of G2-NH_2_ and higher voltages compared to the ones reported earlier (*K_D_* = (7.2 ± 4.7) × 10^−9^ M at *V* = 20 mV) in the multichannel systems were needed because of the increased supporting electrolyte concentrations (1 M vs. 0.1 M) that, by electrostatically screening charges on both the blocker and the channel, weakened blocker binding.

The fast reversible current fluctuation induced by G2-NH_2_ in the parts of current tracks with excluded voltage-dependent gating can be described as a two-state memoryless Markov process, where both the residence time in the blocked state and the channel lifetime in the unblocked state (the time between blockages) are described by exponential distributions. This is demonstrated by the Lorentzian shape of the power spectral density of G2-NH_2_-induced current fluctuations at *f* < 1000 Hz (Figure 2C, spectrum in grey fitted by the smooth blue solid line through the experimental curve). This relatively straightforward kinetic analysis was to a certain extent complicated by a number of factors, namely the two types of complex non-Markovian channel gating described in detail previously [14,15]. The first type of gating is induced by the applied voltage that brings the PA_63_ channel into a nonconductive state, which seems to be characteristic for β-barrel channels in general [38]. This voltage dependent gating was especially prominent at *cis*-side negative voltages [33]; thus applied voltages as low as − (10–20) mV led to the prolonged channel closures. The fact that the β-barrel PA_63_ channel tends to stay closed when positioned under non-physiological inside-negative voltage gradients, adds fuel to the little rusty but still very interesting debate about the significance [39] and mechanism [40] of voltage gating for the unconventional channel [41] function. Note that while some researchers show that the voltage-dependent β-barrel channel closure represents nothing more than an artifact of bilayer lipid experiments [42], others report clear evidence of physiological significance of voltage gating in β-barrel channels [43,44]. In one way or another, this circumstance has largely limited our ability to collect reliable statistics on channel/blocker binding reaction at negative and high positive voltages, especially because in many cases the dendrimer addition has significantly enhanced the voltage sensitivity of the channel. The second type of PA_63_ non-Markov gating is the so-called voltage-independent fast flickering 1/*f* noise between the open and completely closed states that was earlier described as a universal intrinsic property of the pore-forming components of AB type toxins, PA_63_, C2IIa, and Ib, both at the single [2,3,14,15] and multi-channel [8,9] level. The current noise power spectrum of the non-modified PA_63_ channel contains a 1/*f*-like voltage-independent [14,15] component (Figure 2C, see the spectrum in green and the corresponding current track (left insert) shown at 0.2 ms time resolution). Even though the 1/*f* flickering is not among the immediate points of interests of the current publication, the universality of the 1/*f* flickering and the fact that the F427A mutant of PA_63_, which lacks the φ-clamp [7] and therefore A-component translocation functionality, was devoid of the 1/*f* noise behavior, deserves to be studied more closely. Within the limits of this study, we had to be very careful to uncouple the 1/*f* fast-flickering events and the dendrimer-induced reversible blockages, especially under conditions where the closed time distributions of these events partially overlap. For example, in Figure 2A (left) we show several relatively long 1/*f* flickering events that are still seen even at low, 50 ms time resolution. To quantify kinetic parameters of the G2-NH_2_-induced reversible blockages, we primarily used current noise spectral analysis (Figure 2C) instead of the direct counting of open and closed event durations. The direct counting approach does not allow us to distinguish between open times of the 1/*f* noise closures and the dendrimer-induced reversible blockages, because a combined open time distribution for these two processes was single-exponential, which may be explained by the fact that there is only one open state of the channel. The average lifetime of G2-NH_2_ in the channel pore ($t_{res}$) and average time between blockages ($t_{on}$) were found correspondingly as tres=12πfc(1−pbl) and ton=12πfcpbl, where $f_{c}$ is the corner frequency of Lorentzian and $p_{bl}$ is the probability of finding PA_63_ in the blocked state [45]. Ideally, the probability of the channels being in the blocked state could be directly determined as $p_{bl}=\frac{I_{0}-I_{ave}}{I_{0}}$, where $I_{0}$ is the ion current through the completely open channel, and $I_{ave}$ is the average ion channel current modified by the blocker. However here, to account for the 1/*f* fast flickering, the equation was corrected, assuming independence of these two processes as follows: $p_{bl}=\frac{I_{ave}^{free}-I_{ave}}{I_{ave}^{free}}$, where $I_{ave}^{free}$ is the average current through the PA_63_ channel measured in blocker-free solutions [14]. Note: to determine $I_{ave}^{free}$ and $I_{ave}$, the prolonged voltage gating closures (both intrinsic and dendrimer-induced) were excluded from the open and closed states probability analysis.

The second mode of dendrimer-induced current inhibition was hard to describe quantitatively because the blocker-induced channel closures often appeared to be irreversible, lasting for minutes or longer. To reopen the channel, we either had to apply 0 mV or to reverse the voltage sign (shown in Figure 2A, middle) which did not allow us to collect reliable statistics on kinetic parameters of the second mode of channel blockage. Moreover, as described above, the voltage-induced closures were also recorded in the absence of blocker, and voltage-sensitivity and probability of finding a channel in the closed state varied from channel to channel. However qualitatively, this process evinced all the key characteristics of the voltage-induced gating of β-barrel channels, such as strong voltage dependence, prolonged closures (minutes), and difficulties in reopening channels even when voltage was reduced to zero. Paradoxically, channel reopening was often possible with abrupt second-long pulses of high voltages of opposite sign (marked in Figure 2A, middle track), that, in turn, also induced voltage dependent closures if applied longer.

### 2.2. Role of PAMAM Dendrimer Surface Chemistry in PA_63_ Blockage

**Figure 3:**
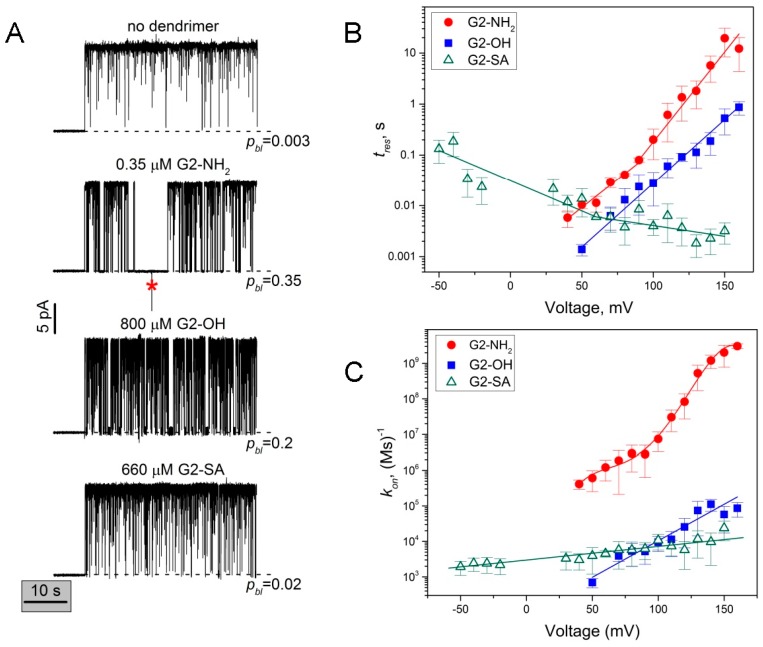
Influence of the PAMAM dendrimer surface chemistry on the PA_63_ single channel current inhibition. (**A**) PA_63_ channel current tracks in the absence (top) and presence of G2-NH_2_, G2-OH, and G2-SA PAMAM dendrimers in the *cis* side of the bilayer chamber (three lower rows). In the dendrimer-free solution, the channel remains open with only 0.3% probability of being in the closed state (*p_bl_*) due to intrinsic 1/*f* noise flickering (mostly filtered). Addition of G2-NH_2_ (second row) induces two modes of PA_63_ current blockage, fast reversible fluctuations and longer voltage gating type of closures (marked by “*”). At 0.35 μM G2-NH_2_, the probability of finding the channel in the closed state increases to 35%. The addition of 800 μM G2-OH (third row) causes fast reversible blockages, whereas the voltage gating events are less pronounced; the probability of finding PA_63_ in the closed state is 20%. G2-SA addition (bottom row) causes reversible blockages at similar sub-mM concentrations, with a 2% probability of finding PA_63_ in the closed state. Applied voltage was 100 mV; current tracks were averaged over a time interval of 10 ms. The dashed lines represent zero current levels; (**B**) Residence times of dendrimer binding reaction plotted as functions of transmembrane voltage. While residence time of G2-OH (filled squares) increases exponentially with voltage (solid line through the data points), residence times of G2-NH_2_ (filled circles) and G2-SA (open triangles) show more complex progression, increasing and decreasing with the voltage increase respectively. The data were split into two voltage intervals and fitted with two separate exponents (solid lines) (**C**) On-rate of dendrimer blockage defined as the inverse of the average open channel life time as a function of voltage for G2-NH_2_ (filled circles), G2-OH (filled squares), and G2-SA (open triangles) blockers. Note the voltage sensitivity of $k_{on}$ for all three dendrimers and the significantly higher absolute values of the on-rate constant in the case of G2-NH_2_.

The common mechanism of inhibiting cation-selective PA_63_ involves blockage of the channel’s lumen by positively charged molecules [2,3,5,7,9,10,14,33,46,47]. However, on a multichannel level in 0.1 M KCl, it has been demonstrated that G2 and G3 PAMAM dendrimers functionalized with surface hydroxyl groups (G2-OH and G3-OH) inhibited PA_63_ channels in a concentration-dependent manner [5]. The *IC*_50_ values were about 20 (Table 1) and 9 times lower compared to those of G2-NH_2_ and G3-NH_2_, respectively. Typical multichannel titration curve analyses are given in Figure S3. Interestingly, even in the presence of PAMAM dendrimer functionalized with negatively charged carboxyl surface group (G1-COONa, but named G0.5 by the manufacturer), some small current decrease was recorded, though compound activity was not high enough to reach 50% inhibitory concentrations. In order to understand how the PA_63_ channel selects among the PAMAM dendrimer blockers, here we investigated kinetic parameters of the binding reaction directly comparing G2-NH_2_, G2-OH, G2-SA, and G2-COONa PAMAM dendrimers binding to PA_63_ on a single channel level (Figure 3 and Figure S4). As before, the single channel measurements are performed in 1 M KCl (Figure 3).

Figure 3A gives four representative recordings of a single PA_63_ channel current modified by different concentrations of G2-NH_2_, G2-OH, G2-SA (three lower rows) compared to dendrimer-free solution (top). When added to the *cis* compartment solutions, all dendrimers reversibly blocked the channel, with *cis*-positive transmembrane voltages significantly enhancing the blockage with G2-NH_2_ and G2-OH, but not G2-SA (Figure 3B,C). Two modes of G2-NH_2_-induced current blockage are evident at a very low sub-µM blocker concentration at 100 mV applied voltage. In the presence of 0.35 µM of G2-NH_2_, probability of finding the channel in the closed state increases to 35% (second row) compared with 0.3% in the dendrimer-free solution (first row). G2-OH dendrimer addition (third row) causes similar complete but reversible channel closures (first mode of blockage), however more than 2000 times higher blocker concentrations were required to increase probability of the closed state to 20%. At voltages ≥100 mV, we also detected the second mode of channel closure (voltage gating events), however this effect was significantly less pronounced compared to the one observed for G2-NH_2_. At comparable sub-mM concentrations and 100 mV, G2-SA, modified with negatively charged surface groups, (fourth row) shows only weak interaction with probability of closed state equal to 2%. We could not detect any concentration-dependent channel fluctuations upon addition of G2-COONa to the *cis* compartment of the bilayer chamber. Instead, high concentration of G2-COONa (3 mM) caused membrane instability and, eventually, breakage (Figure S4).

### 2.3. The Rate Constants of Dendrimer’s First Mode of Binding Reaction are Voltage Dependent

For all three blockers, the on- and off-rate constants of the PA_63_/dendrimer binding reaction varied with the applied voltage (Figure 3B,C). The shown data were obtained with power spectral analysis of the reversible current fluctuations, using the fitting by single Lorentzian spectra as described above (Figure 2C). For voltages ≥130 mV, when strong blocker-induced voltage gating (second type of blockage) prevented us from collecting long current recordings suitable for the power spectral analysis, the data were analyzed by averaging over direct measurements of blocked and open event durations. The on-rate was calculated as kon=1toncPAMAM, where $t_{on}$ is time between successful blockages, and $c_{PAMAM}$ is dendrimer bulk concentration. While in the case of G2-OH, the dendrimer with the positively charged tertiary amine interior and neutral hydroxyl surface groups, $t_{res}$ was shown to increase exponentially (linear fit in the semi-logarithmic scale in Figure 3B), in the case of the “highly” cationic G2-NH_2_ binding, we observed non-exponential voltage dependence of $t_{res}$ (fitted with two linear dependences in Figure 3B). The binding time in the presence of G2-SA, the dendrimer that is made of the positively charged PAMAM core and 16 negatively charged succinamate surface groups, was shown to have inverse voltage dependence, with the $t_{res}$ binding time decreasing with *cis*-positive voltage decrease. Figure 3C shows the on-rate dependence of the PAMAM dendrimer/binding reaction on the applied voltage. Interestingly, for all three dendrimers we observed a voltage-dependent increase in the binding reaction rate constants (Figure 3C), which indicates that high voltages make PAMAM dendrimer capture by the PA_63_ easier. At the same time, $k_{on}$, and, therefore the number of individual blockage events, was drastically higher for G2-NH_2_ compared with both G2-OH and G2-SA.

### 2.4. PA_63_ Blockage by Imperfect Cationic PAMAM Dendrimers

**Figure 4:**
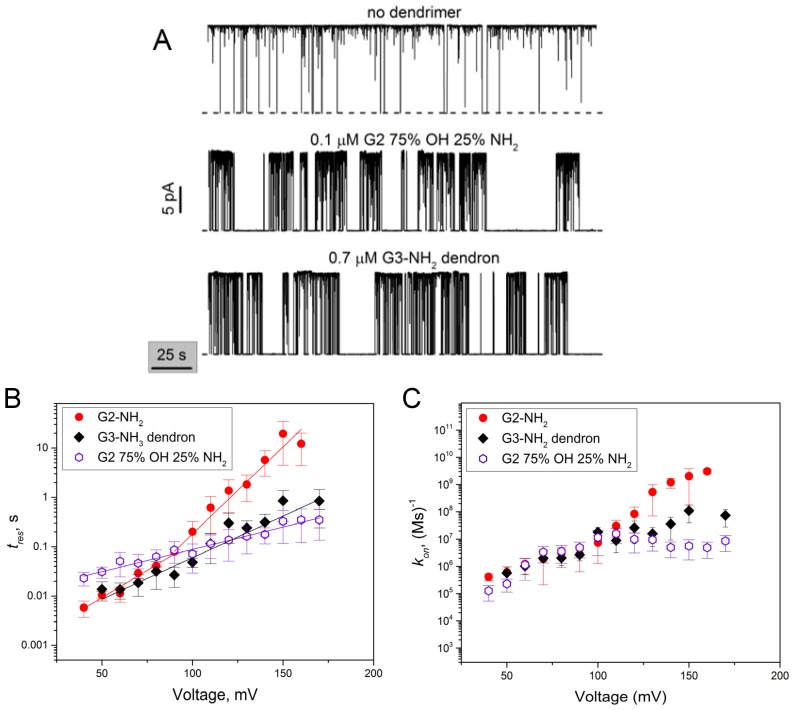
Modulation of a single PA_63_ channel current by imperfect G2-75% OH 25% NH_2_ dendrimer and G3-NH_2_ dendron. (**A**) The sub-μM addition of G2 75% OH 25% NH_2_ dendrimer (middle) and G3-NH_2_ dendron (bottom) to the *cis*-side of the bilayer chamber results in PA_63_ current inhibition. Two modes of the blocker action are clearly seen. In the absence of a blocker (top), the channel is open; the intrinsic 1/*f* flickering events are mostly filtered by averaging over a time interval of 50 ms. Applied voltage was 100 mV, measurements were performed in 1 M KCl. The dashed lines represent zero current levels; (**B**) Residence time of imperfect G2 75% OH 25% NH_2_ dendrimer (open hexagons) and G3 dendron (filled diamonds) binding increases exponentially with voltage increase. Residence time for G2-NH_2_ dendrimer (filled circles) is as discussed in Figure 3B; (**C**) On-rate constant as a function of voltage increases and then shows saturation at *V* > 100 mV in the case of G2 75% OH 25% NH_2_ dendrimer and G3 dendron, but not with G2-NH_2_ where it continues increasing.

Activity of PAMAM dendrimers was previously shown to increase dramatically when they were degraded at the amide linkage, to a heterodisperse population of dendrimers of different molecular weights [20]. These less sterically constrained and hence, more flexible, “imperfect” dendrimers were reported to show significantly enhanced transfection activity compared to the intact dendrimers. To test if a similar effect could be achieved with the pore blockage, we previously investigated the channel blocking activity of two different types of “imperfect” PAMAM dendrimers. First, we tested a mixed-surface G2 75% OH 25% NH_2_ charge-dispersed PAMAM dendrimer, where the proportion of the positively charged surface amino groups was only 25% on average. In 0.1 M KCl, this dendrimer was about 17 times less active against PA_63_ compared to G2-NH_2_. A notable increase in the blocker’s activity was achieved with the structurally incomplete 8+ charged G2 dendron, which was about 26 times more active, compared to the 8+ charged G1-NH_2_ dendrimer [5]. Here we investigate the G2 75% OH 25% NH_2_ dendrimer activity and also use the 16+ charged G3-NH_2_ dendron to directly compare their activity with the 16+ charged G2-NH_2_ dendrimer on a single-channel level (Figure 4). In a manner similar to G2-NH_2_ intact dendrimers, the inhibitive action of the G2 75% OH 25% NH_2_ dendrimer and G3-NH_2_ dendron was bimodal. Both the short reversible blockage events and the prolonged closures were detected after the addition of sub-μM concentrations of either of these two imperfect dendrimers to the *cis*-compartment solutions (Figure 4A). While the effective concentrations were comparable to those used for G2-NH_2_ (and significantly lower compared to G2-OH and G2-SA), the voltage sensitivity of the binding reaction on-rates was substantially different (Figure 4B,C). 

## 3. Discussion

### 3.1. Two Modes of G2-PAMAM Dendrimer Inhibition of PA_63_ Channel

**Figure 5:**
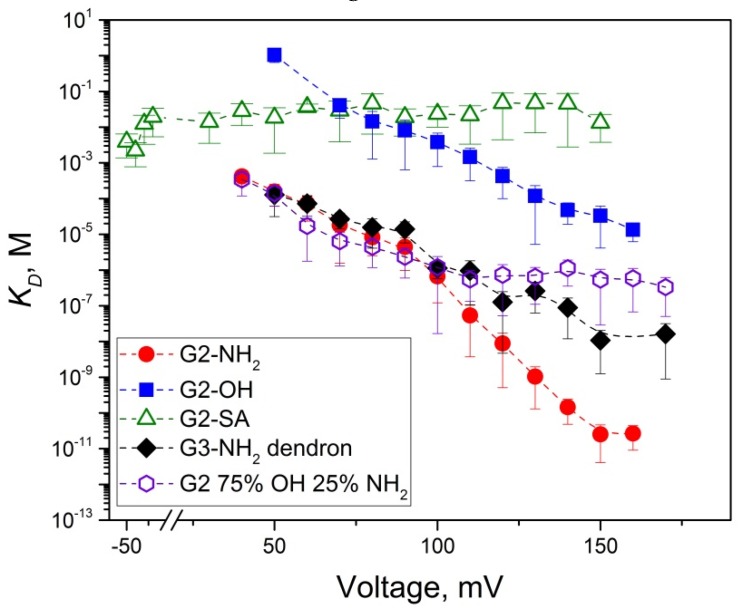
Equilibrium dissociation constants of the PAMAM dendrimer binding reaction to a single PA_63_ pore plotted as a function of applied transmembrane voltage. Note that only first mode of blockage type events were considered. *K_D_* values significantly decrease with voltage (stronger binding) for all the shown blockers, except G2-SA. The G2-OH dendrimer *K_D_* values are significantly higher (less effective binding) compared to those for G2-NH_2_, G2 75% OH 25% NH_2_ dendrimers and the G3-NH_2_ dendron. The experiments were performed in 1 M KCl at pH 6.

In this paper, we used the single channel planar lipid bilayer technique to present evidence that second generation PAMAM dendrimers inhibit the PA_63_ channel of anthrax toxin by ion current blockage. For all tested G2 PAMAM dendrimers and the G3-NH_2_ PAMAM dendron, the channel current inhibition was bimodal. The first mode of the ion current inhibition was observed in the form of complete (100% of total channel conductance) but reversible ion current fluctuations that were described by a two-state Markov process, with one state being an open, dendrimer-free state and second state being a blocked, dendrimer-bound state. Interestingly, not only the three tested cationic blockers (intact G2-NH_2_, mixed surface 75% OH 25% NH_2_ dendrimers, and G3-NH_2_ dendron) but also G2-OH and G2-SA dendrimers (functionalized, respectively, with neutral and negatively charged terminal groups) reversibly blocked the K^+^ current through PA_63_, apparently physically entering the channel’s permeation pathway. Figure 5 summarizes the equilibrium dissociation constants for all five blockers that were calculated using the blocker/channel binding reaction kinetic constants shown in Figure 3 and Figure 4 as $K_{D}=\frac{k_{off}}{k_{on}}$, where $k_{off}=\frac{1}{t_{res}}$. It can be seen (Figure 5) that G2-NH_2_ activity significantly exceeds (lower *K_D_* values) that of G2-OH and G2-SA, which is mostly determined by the dramatically higher on-rates of the G2-OH/PA_63_ and G2-SA/PA_63_ binding reactions rather than by the reactions’ off-rates. Thus, under 50 mV of applied voltage, $t_{res}$ of G2-OH was about 10 times lower, and $t_{res}$ of G2-SA was comparable with that of G2-NH_2_ (Figure 3B). The on-rates were about three-fold and two-and-a-half-fold higher for G2-NH_2_ compared with G2-SA and G2-OH, respectively (Figure 3C). In contrast, the on-rate of the binding reaction was nearly identical when G2 75% OH 25% NH_2_ dendrimer and G3-NH_2_ dendron were investigated and compared with G2-NH_2_ dendrimer at 50 mV (Figure 4C).

In addition to the reversible two-state Markov process blockage events, all five blockers caused long voltage-dependent PA_63_ closures that, in many aspects, resembled the typical characteristics of the classical voltage gating of β-barrel ion channels [42] (Figure S2). The quasi-irreversible character of the voltage gating allowed us to perform only qualitative analysis of this type of blocker-induced closure. In a manner similar to the reversible Markov PA_63_ blockage process, the voltage gating type of closures appeared to be stronger when the dendrimers were decorated with positively-charged terminal groups and was very weak in the case of G2-SA (see e.g., Figure 3A where no voltage dependent closures are seen in the presence of 660 µL of G2-SA over ~50 s recording). Previously, we described the two similar modes of the blocker-induced channel inhibition with the 7-fold symmetrical β-cyclodextrin inhibitors [14,15]. Because the molecular mechanism, possibly a universal one, of the voltage gating observed for many functionally distinct β-barrel channels in planar lipid bilayers has yet to be clarified [40] and its physiological relevance has been called in question [39], we cannot make a strong judgement on the biological importance of the second mode of the dendrimer-induced PA_63_ current inhibition. However, we want to emphasize that when we attempted to study the dendrimer and cyclodextrin binding reaction single-channel kinetics at low, close to physiological salt concentrations (e.g., in 0.1 M solutions), the second type of the current inhibition was very strong. This led to prolonged and complete channel closures at very low, (20–50) mV applied voltages, precluding us from performing kinetic investigation of the PA_63_/multivalent blocker binding reaction. For the same reason, we had difficulties studying the first type of the blockages at voltages >(150–180) mV, because the quasi-irreversible voltage gating events were observed at extremely low blocker concentrations (0.01–0.1 nM) when the number of the first type of reversible blockage events was too low compared to the natural 1/*f* current fluctuations of this very complex channel. Previously we reported a reasonably good linear correlation (*R* = 0.84) between activity of the PA_63_ channel cyclodextrin inhibitors in RAW 264.7 cells and in the multichannel reconstitution assays [48], allowing us to suggest that the second voltage gating type of closures is physiologically relevant. Moreover, the strong asymmetry that we and others observed in non-modified PA_63_ voltage gating with the voltage sign (the channel tends to close even at very low *cis*-negative voltages) [33,49], suggests that the voltage gating could be an internal tool for PA_63_ to stay closed when being occasionally inserted into off-target bilayers.

Note that the *K_D_* data in Figure 5, when approximated to 20 mV, are higher compared with the equilibrium *IC*_50_ values measured at 20 mV (Table 1). All the blockers appear to be more effective when used at the multichannel level. The explanation of this apparent discrepancy is simple and identical to the one previously suggested for the PA_63_/7+β-CD binding reaction [14]. The *IC*_50_ values measured at the multichannel level contain both the fast reversible dendrimer-induced blockage events (first mode of dendrimer action) and the prolonged voltage gating closures (second mode of action), whereas the voltage gating events were intentionally excluded from binding reaction kinetics analysis at the single channel level. As discussed before, we believe that the second mode of PA_63_ current inhibition is related to the voltage gating of β-barrel channels, well-known to any electrophysiologist who works with these channels. It appears that β-CD and dendrimer addition significantly lowers the so-called “critical voltage” needed for the channel gating. Because we currently lack understanding of the voltage gating mechanism, we cannot comment on the physiological relevance of this second mode of current blockage (frequently a more intense one) in anthrax toxin inhibition. Note that the β-CD and dendrimer blockers were previously shown to effectively protect cells [2,3] and animals [50] against the anthrax toxin.

### 3.2. Voltage Dependence of the Reversible Dendrimer/PA_63_ Interaction

With all previously tested β-cyclodextrin PA_63_ blockers, nearly exponential (linear in semi logarithmic scale) voltage dependence of $t_{res}$ was reported with almost identical voltage sensitivity between two tested 7+ charged β-CD blockers, whereas the $k_{on}$ values were only weakly voltage-dependent [14,15]. Moreover, it was the off-rate and not the on-rate that mainly determined the earlier reported difference in 7+β-CD potency. Details of the mechanism of the PAMAM dendrimer-induced PA_63_ current blockage are different. Thus in the case of G2-OH, the dendrimer with a positively charged tertiary amine interior and neutral hydroxyl surface groups, $t_{res}$ was shown to increase exponentially with voltage (linear fit in the semi-logarithmic scale in Figure 3B, filled squares) with the slope of the logarithm of the lifetimes versus voltage dependence $\mathrm{dlg}t_{res}/dV=(25\pm 0.6)\times 10^{-3}\;{\left(\mathrm{mV}\right)}^{-1}$. In contrast, with the “highly” cationic (16 terminal amino groups) G2-NH_2_, we observed a non-exponential $t_{res}$ increase with voltage. The $t_{res}$ voltage dependence data were broken into two intervals and each was fitted with a single exponent (shown as two linear dependences in the semi-logarithmic scale in Figure 3B, filled circles). The slopes of the logarithm of the lifetimes versus voltage dependence for low and high voltages were: $\mathrm{dlg}t_{res}/dV=(22\pm 2)\times 10^{-3}\;{\left(\mathrm{mV}\right)}^{-1}$ at low *cis*-positive voltages and $\mathrm{dlg}t_{res}/dV=(35\pm 2)\times 10^{-3}\;{\left(\mathrm{mV}\right)}^{-1}$ at high *cis*-positive voltages. The binding time in the presence of G2-SA, the dendrimer that is made of the positively charged PAMAM core and 16 negatively charged succinamate surface groups, was shown to have inverse voltage dependence, with the $t_{res}$ decreasing with *cis*-positive voltage decrease. The G2-SA residence time voltage dependence was also approximated with two separate single exponential dependences (Figure 3B, open triangles), one at lower voltages ($\mathrm{dlg}t_{res}/dV=-(12\pm 1)\times 10^{-3}\;{\left(\mathrm{mV}\right)}^{-1}$) and another at higher voltages ($\mathrm{dlg}t_{res}/dV=-(4.2\pm 0.9)\times 10^{-3}\;{\left(\mathrm{mV}\right)}^{-1})$. It appears that the high *cis*-positive and high *cis*-negative applied voltages increase voltage sensitivity of the PA_63_/G2-NH_2_ and PA_63_/G2-SA binding reactions, respectively. Thus, the $t_{res}$/voltage dependence data could be used to determine the so-called effective “gating charge” [51], a parameter characterizing sensitivity of the blockage reaction to voltage [52], as $\delta z=k_{B}T\frac{d2.303\mathrm{lg}t_{res}}{dV}$. Here, δ is the dimensionless “apparent electrical distance” to the blocking site, z is the blocker charge, V is the applied voltage, and $k_{B}$ and T have their usual meaning as the Boltzmann constant and absolute temperature (in degrees Kelvin), respectively. The G2-OH binding could be characterized by δz=1.50±0.03 elementary charges, the G2-NH_2_ binding by δz=1.3±0.2 (low voltage) and δz=2.10±0.07 (high voltage) elementary charges, and G2-SA binding by δz=−0.72±0.08 (low voltage) and δz=−0.25±0.05 (high voltage) elementary charges. The twisted residence time voltage dependence observed with G2-NH_2_ and G2-SA but not with G2-OH could tentatively be explained by structural reorientation of the terminal amino and succinamate groups or solvation structure (shell water and counterions) reorganization under the applied electrostatic field. Interestingly, $k_{on}$ increases as a function of voltage (Figure 3C) showing that high voltages facilitate dendrimer delivery to the binding site, in contrast to the very weak voltage dependence of the binding reaction on-rate reported earlier for 7+β-CDs but in a manner similar to that reported for alpha-synuclein/α-hemolysin binding reaction [53]. Surprisingly, we also observed a slight increase in the on-rate with *cis*-positive voltage increase even for G2-SA dendrimer, which carries 16 negatively-charged terminal groups.

We have also investigated the role of the so-called “imperfect” dendrimers in the PA_63_ blockage dynamics using G2 75% OH 25% NH_2_ dendrimer which, on average, has only four terminal positive charges and G3-NH_2_ dendron decorated with 16 positively-charged terminal groups. The residence times of the blockers inside the channel changed exponentially with voltage showing more shallow slopes compared with those earlier discussed for G2-NH_2_: $\mathrm{dlg}t_{res}/dV=(16.8\pm 1.5)\times 10^{-3}\;\left(\mathrm{mV}\right)$^−1^ for the G3-NH_2_ dendron and $\mathrm{dlg}t_{res}/dV=(9.1\pm 0.3)\times 10^{-3}\;\left(\mathrm{mV}\right)$^−1^ for G2 75% OH 25% NH_2_ dendrimer (Figure 4B). These two systems can be characterized by the effective “gating charge” δz=0.92±0.16 (G2-NH_2_ dendron) and δz=0.53±0.03 (G2 75% OH 25% NH_2_) elementary charges showing weaker residence time voltage sensitivity compared to G2-NH_2_. At the same time at voltages <80 mV, G3-NH_2_ dendron lifetime is comparable and G2 75% OH 25% NH_2_ lifetime is even higher than that for G2-NH_2_ (Figure 4C). At *V* < 120 mV the binding reaction on-rates are comparable for all three blockers, however at *V* > 120, $t_{on}$ shows only weak voltage dependence for both the imperfect dendrimers.

## 4. Conclusions

Since their discovery in 1985 by Tomalia [54,55], PAMAM dendrimers have been the subject of thorough theoretical and experimental investigation in soft-matter physics, not only due to their seemingly high commercial potential but also because of their unique “ultrasoft colloid” properties bridging the gap between polymers and hard spheres [56]. PAMAM dendrimer conformational flexibility in solutions and the effect of solvent and pH on their structure, swelling, charge, counterion distribution, degree of protonation, and deformability have been addressed in a significant number of publications [57,58,59,60,61,62,63,64,65,66,67,68,69,70,71]. At the same time, single channel studies investigating specifics of the dendrimer dynamics in ion channel confinement are limited. In 2000, a rapid nuclear pore sizing patch-clamp method based on the calibrated fluorescently-labeled amino-terminated dendrimers was described [72]. In 2007, sulfhydryl-reactive poly(amido amine) G2, G3, and G5 dendrimers of second, third and fifth generations decorated with a mixed surface of terminal hydroxyl and amine groups were designed to interact with α-hemolysin channels that contained engineered cysteine residues [73], with the ultimate goal of modifying the stochastic-sensing properties of α-hemolysin upon addition of the charged and dense dendrimers into its lumen. The dendrimers acted as both an ion-selectivity filter and a molecular sieve, regulating the passage of small- and macromolecules. In 2013, polypropylenimine dotriaconta-amine G3 and G4 dendrimers were tested against the *E. coli* E69 pore-forming Wza K30 capsular polysaccharide transporter; however, no detectable inhibitory activity was reported [74]. In 2014, PAMAM dendrimers were reported to effectively block the ion-channel forming components of the anthrax and C2 toxins using planar lipid bilayer measurements and cell assays [5]. More recently, α-hemolysin/PAMAM dendrimer system was used to investigate the molecular process of ion channel confinement and its effect on dendrimer conformation using single channel measurements and molecular dynamics simulations [75]. The authors have shown that the electrophoretic migration of the polycationic dendrimers into a confined space is determined by the generation-dependent compressibility of the dendrimers rather than by their diameter. The ion channel nanoscale confinement had also reduced the PAMAM dendrimer protonation. Just recently, fully atomistic molecular dynamics simulations were used to investigate pH-dependent blockage (the authors call it “gating”) of the cytolysin A pore by PAMAM dendrimers [76]. The protonated dendrimers were able to adapt a more extended conformation, effectively blocking about 91% of the channel current, whereas the non-protonated dendrimers were more compact, which created some void space for water and ion passage and about 31% reduction in current.

In this study, we investigated the effect of the PAMAM dendrimer surface chemistry and structural integrity on their ability to enter and block the ion (and probably lethal and edema factor) translocation pathway of the anthrax toxin channel. Considering the electrostatic nature of the cationic blocker interaction with the strongly cation-selective PA_63_ pore, it comes as no surprise that the residence time of the dendrimer/channel binding reaction turned out to be dependent on both blocker chemistry and transmembrane voltage. One of the unforeseen findings made in this study is the increase in the dendrimer capture rate (the binding reaction on-rate) with the *trans*-negative voltage increase and the strong on-rate dependence on dendrimer surface chemistry. Indeed, the on-rate, which is proportional to the number of effective binding events, is traditionally believed to be determined by a correlation between the particle size and the channel entry area (or squared radius). The applied electrical field that falls primarily across the channel (traditionally, across the membrane) is not expected to significantly influence the capture kinetics at distances greater than 3 Å away (1 Debye length in 1 M KCl) from the channel entrance. Thus, previously reported strong voltage sensitivity of a particle capture for the tubulin/VDAC [77] and alpha-synuclein/α-hemolysin [53] binding reactions was explained by the tubulin and alpha-synuclein interaction with the bilayer lipid membranes as an essential first step before the channel lumen binding. This explanation is clearly not appropriate for dendrimer/PA_63_ binding because of its compact nature. The size of the blocker (29 Å) and extremely elongated structure of the PA_63_ channel [34] means that it may extend to a distance greater than 100 Å above the membrane surface. Nevertheless, PAMAM dendrimers were previously shown to interact with the bilayer lipid membranes [78,79], and this therefore leaves room for the possible membrane-binding related effects on the ion channel conductance. For example, the blocker-induced PA_63_ voltage gating (the second mode of action), shown to depend on both the multivalent blocker [5] addition and the bilayer lipid composition [14], may originate from the dendrimer/membrane interaction process. At the same time, with the exception of G2-COONa (Figure S4), we did not observe any significant membrane instability upon PAMAM dendrimer addition to the membrane bathing solutions.

## 5. Materials and Methods

### 5.1. Reagents

PA_63_ was purchased from List Biological Laboratories, Inc., (Campbell, CA, USA). The following chemical reagents were used: KCl, MES, KOH, and HCl (Sigma-Aldrich, St. Louis, MO, USA), “purum” hexadecane (Fluka, Buchs, Switzerland), diphytanoylphosphatidylcholine, (DPhPC, Avanti Polar lipids, Inc., Alabaster, AL, USA), pentane (Burdick and Jackson, Muskegon, MI, USA), and agarose (Bethesda Research Laboratory, Gaithersburg, MD, USA). MQ water was used to prepare solutions. Primary amine (generation 2) and hydroxyl (generation 2) PAMAM dendrimers, commercially available at Dendritech, Inc., (Midland, MI, USA) as *w*/*w* H_2_O solutions, were a kind gift from Dr. Sergey Bezrukov. G3 primary amino dendrons, mixed-surface 75% OH 25% G2-NH_2_ dendrimers, G2 carboxylate-Na terminated PAMAM dendrimers and G2 succinamic acid terminated PAMAM dendrimers were purchased from Dendritech, Inc. (Midland, MI, USA) as *w*/*w* H_2_O solutions. Note that G2-COONa dendrimers were named G1.5 by the manufacturer because instead of only the terminal amino groups (like in the case of G2-NH_2_ vs. G2-OH substitution), all –NH–CH_2_–CH_2_–NH_2_ end groups were replaced with the COONa substituents, shortening the terminal chain of each branch by about a half. Dendritech, Inc has provided us with the analytical measurements on their PAMAM dendrimer products which are given in Figures S5 and S6. However even though mass spectrometry is often considered as the main method to characterize the presence and nature of defects in the dendrimer structure, it was demonstrated that “dendrimer purity needs to be interpreted with care and may be misleading in the sense that falsely negative results are obtained” [80].

### 5.2. Channel Reconstitution into Planar Lipid Bilayers

To form solvent-free planar lipid bilayers with the lipid monolayer opposition technique [81], we used a 5 mg/mL stock solution of diphytanoylphosphatidylcholine (DPhPC) in pentane. Bilayer lipid membranes were formed on a 60-μm-diameter aperture in the 15-μm-thick Teflon film that separated the electrolyte chamber into two compartments, as described in detail elsewhere [14]. The 0.1 and 1 M aqueous solutions of KCl were buffered at pH 6 (5 mM MES) at room temperature (23 ± 0.5 °C). Single channels were formed by adding 0.5 to 1 μL of 20 μg·mL^−1^ solution of PA_63_ to the 1.5 mL aqueous phase in the *cis*-half of the bilayer chamber. Under this protocol, PA_63_ channel insertions were always directional, as judged by channel conductance asymmetry in the applied transmembrane voltage. Multichannel experiments were performed in 0.1 M and 1 M KCl solutions, buffered at pH 6 by 5 mM MES, at 20 mV applied voltage; ∼1−2 μL of 1 mg·mL^−1^ stock PA_63_ solution was added to the *cis*-side of the chamber. The electrical potential difference across the lipid bilayer was applied with a pair of Ag-AgCl electrodes in 2 M KCl, 1.5% agarose bridges. In all experiments, the PAMAM dendrimers were added to the *cis*-compartment of a bilayer chamber, which was the side of PA_63_ addition. The *cis* compartment is believed to correspond to the endosome-facing “flower” side of the channel. Single-channel measurements were performed at −50 to +180 mV. Multichannel experiments were performed at 20 mV. The applied potential is defined as positive if it is higher on the side of protein addition (*cis*-side). Conductance measurements were done using an Axopatch 200B amplifier (Molecular Devices, LLC., Sunnyvale, CA, USA) in the voltage clamp mode. Signals were filtered by a low-pass 8-pole Butterworth filter (Model 9002, Frequency Devices, Inc., Haverhill, MA, USA) at 15 Hz for multichannel and 15 kHz for single channel systems and sampled with a frequency of 50 Hz and 50 kHz for multichannel and single channel experiments respectively. Amplitude, lifetime, and fluctuation analysis was performed with ClampFit 10.2 (Molecular Devices, Sunnyvale, CA, USA) and OriginPro 8.5 (OriginLab, Northampton, MA, USA) software, as well as with software developed in-house.

### 5.3. Reproducibility of the Experiments and Statistics

All multichannel planar lipid measurements, performed to obtain the *IC*_50_ data shown in Table 1, were repeated at least two times. Values are given as the means ± standard deviations. The single-channel statistical analysis with G2-NH_2_, G2-OH, G2-SA dendrimers and G3 dendron was performed with the power spectral analysis of hundreds of dendrimer-induced current fluctuation events. At voltages >150 mV, because of the strong intrinsic and blocker-induced voltage gating, collection of a significant number of the reversible binding events proved to be difficult. Therefore the on- and off-rates were determined by averaging over lifetimes of several dozen events. Because of the wide distribution of lifetimes in the case of non-homogeneous G2 75% NH_2_ 25% OH dendrimer, the recordings were analyzed by averaging over the event lifetimes at all voltages. The standard deviation for $t_{res}$ (${\mathrm{SD}}_{t_{res}}$) and $k_{on}$ (${\mathrm{SD}}_{k_{on}}$) were then determined by averaging over 2–6 recordings taken independently from different PA_63_ channel reconstitution experiments. The standard deviations for *K_D_* values were calculated from SDs determined for the on- and off-rates as ${\mathrm{SD}}_{K_{D}}=\frac{1}{t_{res}k_{on}}\sqrt{{\left(\frac{{\mathrm{SD}}_{t_{res}}}{t_{res}}\right)}^{2}+{\left(\frac{{\mathrm{SD}}_{k_{on}}}{k_{on}}\right)}^{2}}$.

## Figures and Tables

**Table 1:** Inhibition of PA_63_ channel ion current by intact G2 PAMAM dendrimers and G3 PAMAM dendron expressed as experimental *IC*_50_ values.

	PA_63_/PAMAM Dendrimer Binding Reaction, *IC*_50_					
	G2-NH_2_	G2-OH	G2-SA	G2-COONa	G2 75% OH 25% NH_2_	G3-NH_2_ Dendron
0.1 M KCl	7.2 ± 4.7 nM	142 ± 36 nM	879 ± 50 µM	>14 mM	122 ± 35 nM	16.4 ± 4.0 nM
1 M KCl	5.1 ± 2.6 mM	>30 mM	1.7 ± 0.2 mM	not determined	7.7 ± 0.2 mM	7.8 ± 1.0 mM

All data were calculated as means from at least two separate multichannel experiments; the errors are standard deviations. 0.1 M and 1 M KCl solutions at pH 6 were buffered by 5 mM MES. Recordings were taken at 20 mV applied voltage, which was *cis*-side positive. Note: because G2-COONa activity in 0.1 M KCl was too low to reliably detect *IC*_50_, we did not attempt to perform experiments in 1 M KCl bathing solutions, where charges on both the channel and the dendrimer were to a certain extent screened by the bathing solution counterions.

## Supplementary Materials

The following are available online at www.mdpi.com/2072-6651/8/11/337/s1, Figure S1, Chemical structures of the PAMAM dendrimers used in this study. (A) Cationic PAMAM dendrimers G2-NH_2_, with 16 positively charged terminal groups (left), G2-OH, with positively-charged PAMAM core and neutral OH terminal groups (right). (B) G2 PAMAM dendrimers with negatively charged succinamate (left) and carboxyl (right) terminal groups, G2-SA and G2-COONa respectively. (C) Imperfect G2 PAMAM dendrimers G2 75% OH 25% NH_2_, with 12 neutral OH and 4 positively charged NH_2_ terminal groups on average (left), and G3-NH_2_ dendron, with a fractured more flexible structure and 16 positively charged terminal groups (right). Similar to the Figure 1B color coding, terminal primary amines are colored in red; core tertiary amines are colored in green; terminal hydroxyl groups are colored in blue. The images were created using chemical drawing software ChemDoodle 8.1.0, iChemLabs, LLC. Note that in contrast to all other dendrimers, G2 75% OH 25% NH_2_ is not monodisperse and contains 75% of terminal OH groups and 25% of terminal NH_2_ groups on average. Figure S2, Second mode of G2-NH_2_-induced modulation of a single PA_63_ channel current. At the relatively low applied voltages (70 and 80 mV), PA_63_ mostly remains in an open state in the blocker-free solutions (left, two upper rows). Fast flickering between the open and closed states (the so-called 1/*f* noise) is mostly removed by averaging over a time interval of 100 ms. At 90 mV (left, low row), several pronounced voltage gating events are seen; *p_bl_* = 0.12. In the presence of 0.35 μM of G2-NH_2_ (right), the voltage gating of the channel is significantly increased. Multiple fast current blockages (first mode of dendrimer-induced current inhibition) are observed but they are partially filtered over a time interval of 100 ms. Figure S3, Influence of the PAMAM dendrimer terminal group chemistry on the PA_63_ channel inhibition studied on a multichannel level. (A) A typical dendrimer-induced PA_63_ inhibition curve (shown for G2-OH dendrimer). G2-OH additions are marked with the downward arrows; total bulk dendrimer concentration is indicated. The dashed line represents zero current level; (B) Typical multichannel titration curves of the PA_63_ channel inhibition by G2-NH_2_, G2-OH, and G2-SA dendrimers. The dashed line represents 50% of the original current level. The recordings were taken in 0.1 M KCl solutions at pH 6 under 20 mV applied voltage. Figure S4, Effect of G2-COONa *cis*-side addition on a single PA_63_ channel. Both in blocker-free solution and in presence of 3 mM G2-COONa, PA_63_ mostly remains in an open state. The 1/*f* events are to a large extent removed by averaging over a time interval of 10 ms. G2-COONa addition causes lipid bilayer instability (upward events) and, eventually, breakage. Recordings were taken in 1 M KCl solutions at pH 6 and 100 mV applied voltage. Figure S5, MALDI-TOF mass spectra of G2-NH_2_ (A); G2-OH (B); G2-SA (C); G2-COONa (D) dendrimers and G3-NH_2_ dendron (E). The data were provided by Dendritech, Inc. (Midland, MI, USA). Figure S6, Characterization of generation 2 PAMAM dendrimers and generation 3 PAMAM-NH_2_ dendron by 13 C NMR. The data were provided by Dendritech, Inc. (Midland, MI, USA), (A) 13 C NMR of G2-NH_2_. (B) 13 C NMR of G2-OH; (C) 13 C NMR of G2-SA; (D) 13 C NMR of G2-COONa; (E) 13 C NMR G3-NH_2_ dendron.
