# Supplementary material for: Impact of Dendrimer Terminal Group Chemistry on Blockage of the Anthrax Toxin Channel: A Single Molecule Study
Source: Toxins (Basel). 2016 Nov 15;8(11):337. doi: 10.3390/toxins8110337 (PMC5127133; doi:10.3390/toxins8110337)
Supplement: Supplementary file 1 [file toxins-08-00337-s001.pdf]

Supplementary Materials: Impact of Dendrimer Terminal Group Chemistry on Blockage of the Anthrax Toxin Channel: A Single Molecule Study

Goli Yamini, Nnanya Kalu and Ekaterina M. Nestorovich

Cationic PAMAM dendrimers

A

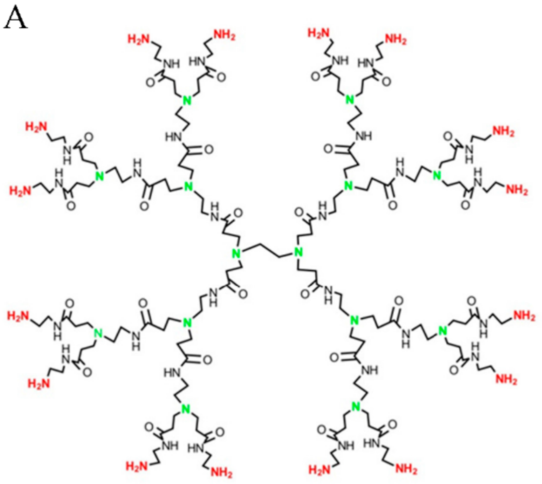

G2-NH$_2$

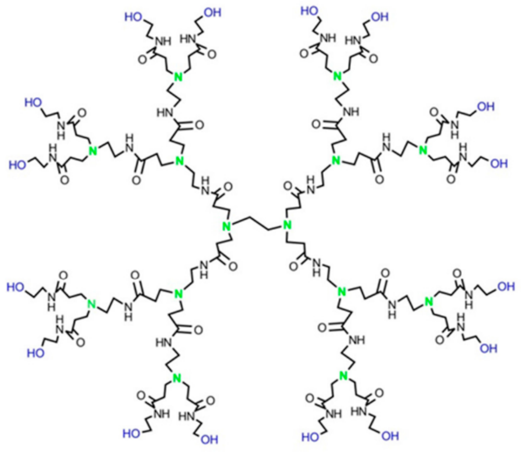

G2-OH

G2 PAMAM dendrimers with negatively charged terminal groups

B

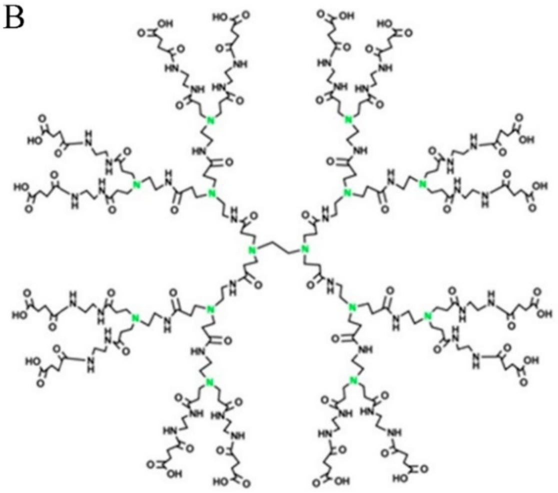

G2-SA

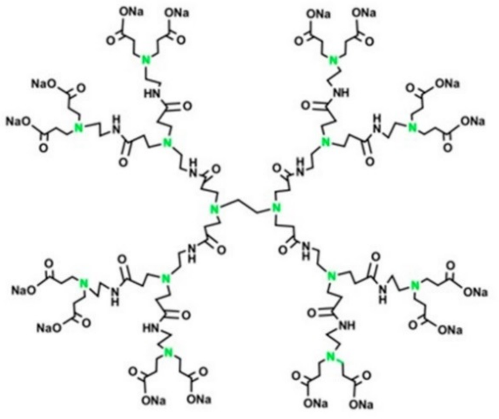

G2-COONa

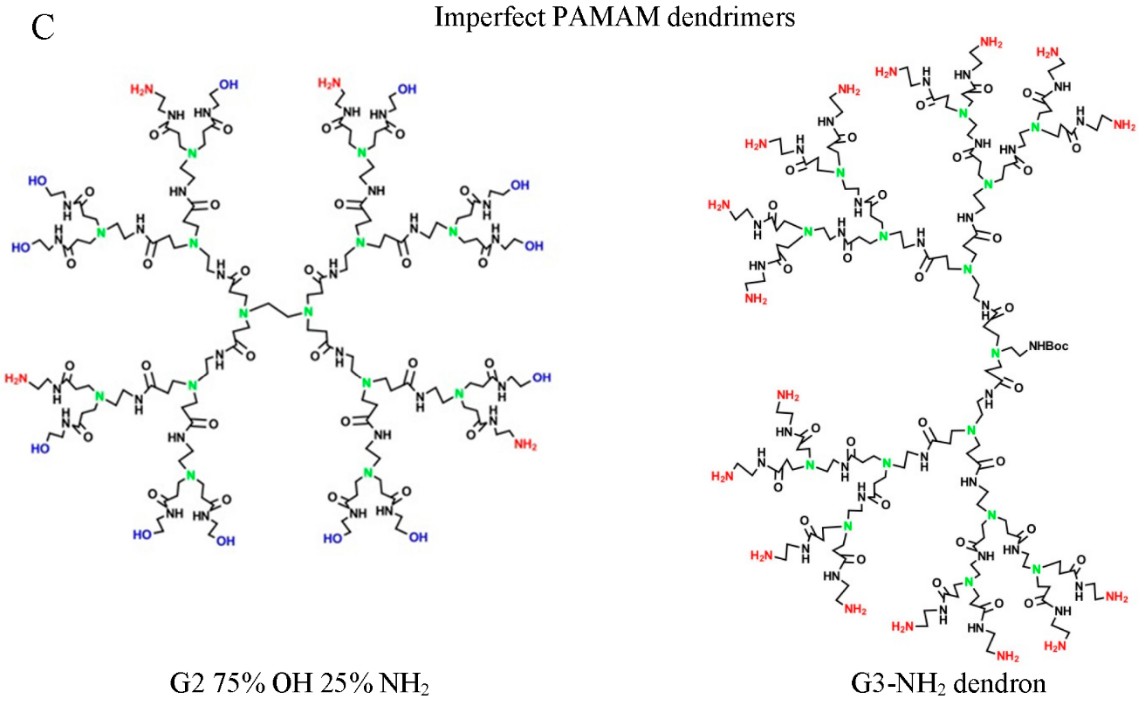

Figure S1. Chemical structures of the PAMAM dendrimers used in this study. (**A**) Cationic PAMAM dendrimers G2-NH_2, with 16 positively charged terminal groups (left), G2-OH, with positively-charged PAMAM core and neutral OH terminal groups (right). (**B**) G2 PAMAM dendrimers with negatively charged succinamate (left) and carboxyl (right) terminal groups, G2-SA and G2-COONa respectively. (**C**) Imperfect G2 PAMAM dendrimers G2 75% OH 25 % NH_2, with 12 neutral OH and 4 positively charged NH_2 terminal groups on average (left), and G3-NH_2 dendron, with a fractured more flexible structure and 16 positively charged terminal groups (right). Similar to the Figure 1B color coding, terminal primary amines are colored in red; core tertiary amines are colored in green; terminal hydroxyl groups are colored in blue. The images were created using chemical drawing software ChemDoodle 8.1.0, iChemLabs, LLC. Note that in contrast to all other dendrimers, G2 75% OH 25% NH_2 is not monodisperse and contains 75% of terminal OH groups and 25% of terminal NH_2 groups on average.

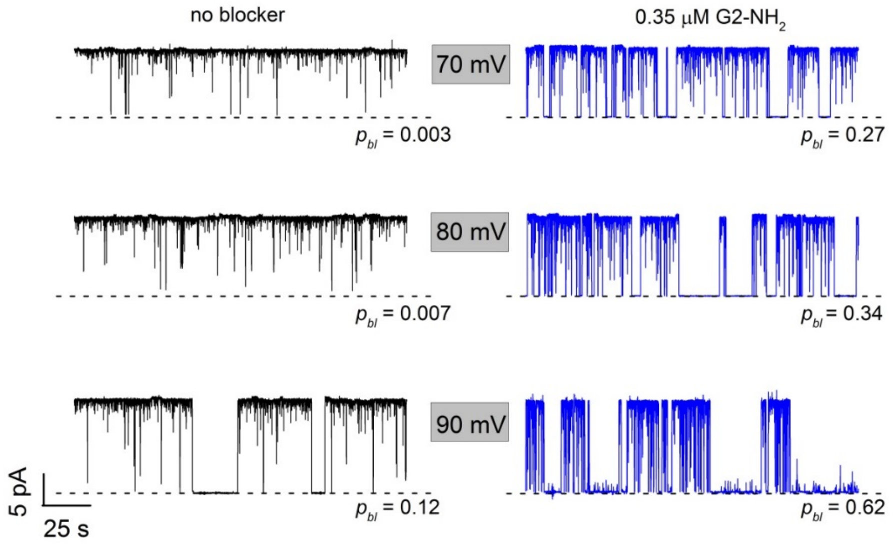

Figure S2. Second mode of G2-NH_2-induced modulation of a single PA_{63} channel current. At the relatively low applied voltages (70 and 80 mV), PA_{63} mostly remains in an open state in the blocker-free solutions (left, two upper rows). Fast flickering between the open and closed states (the so-called

1/*f* noise) is mostly removed by averaging over a time interval of 100 ms. At 90 mV (left, low row), several pronounced voltage gating events are seen; p_{bl} = 0.12. In the presence of 0.35 μM of G2-NH2 (right), the voltage gating of the channel is significantly increased. Multiple fast current blockages (first mode of dendrimer-induced current inhibition) are observed but they are partially filtered over a time interval of 100 ms.

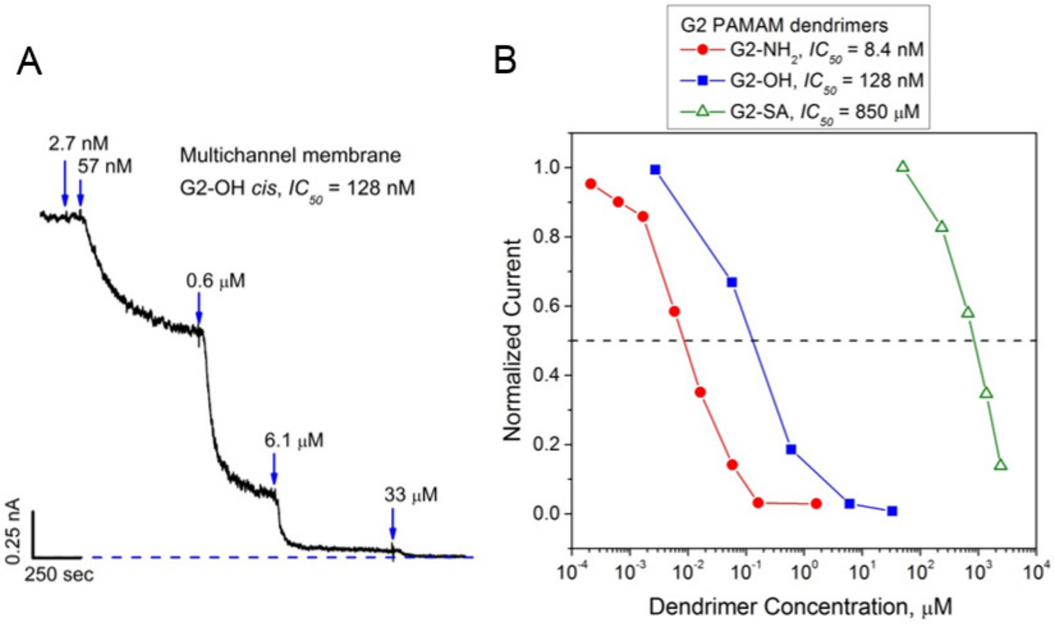

Figure S3. Influence of the PAMAM dendrimer terminal group chemistry on the PA63 channel inhibition studied on a multichannel level. (**A**) A typical dendrimer-induced PA63 inhibition curve (shown for G2-OH dendrimer). G2-OH additions are marked with the downward arrows; total bulk dendrimer concentration is indicated. The dashed line represents zero current level; (**B**) Typical multichannel titration curves of the PA63 channel inhibition by G2-NH2, G2-OH, and G2-SA dendrimers. The dashed line represents 50% of the original current level. The recordings were taken in 0.1 M KCl solutions at pH 6 under 20 mV applied voltage.

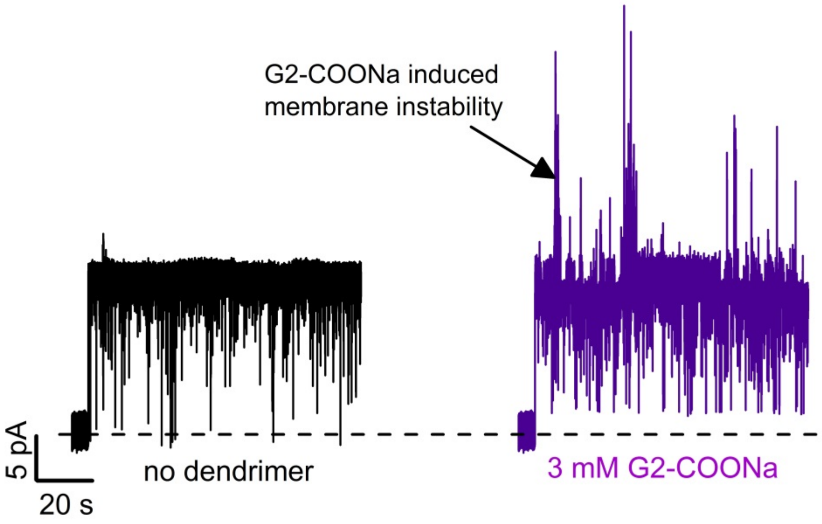

Figure S4. Effect of G2-COONa *cis*-side addition on a single PA63 channel. Both in blocker-free solution and in presence of 3 mM G2-COONa, PA63 mostly remains in an open state. The 1/*f* events are to a large extent removed by averaging over a time interval of 10 ms. G2-COONa addition causes lipid bilayer instability (upward events) and, eventually, breakage. Recordings were taken in 1 M KCl solutions at pH 6 and 100 mV applied voltage.

A G2-NH_2

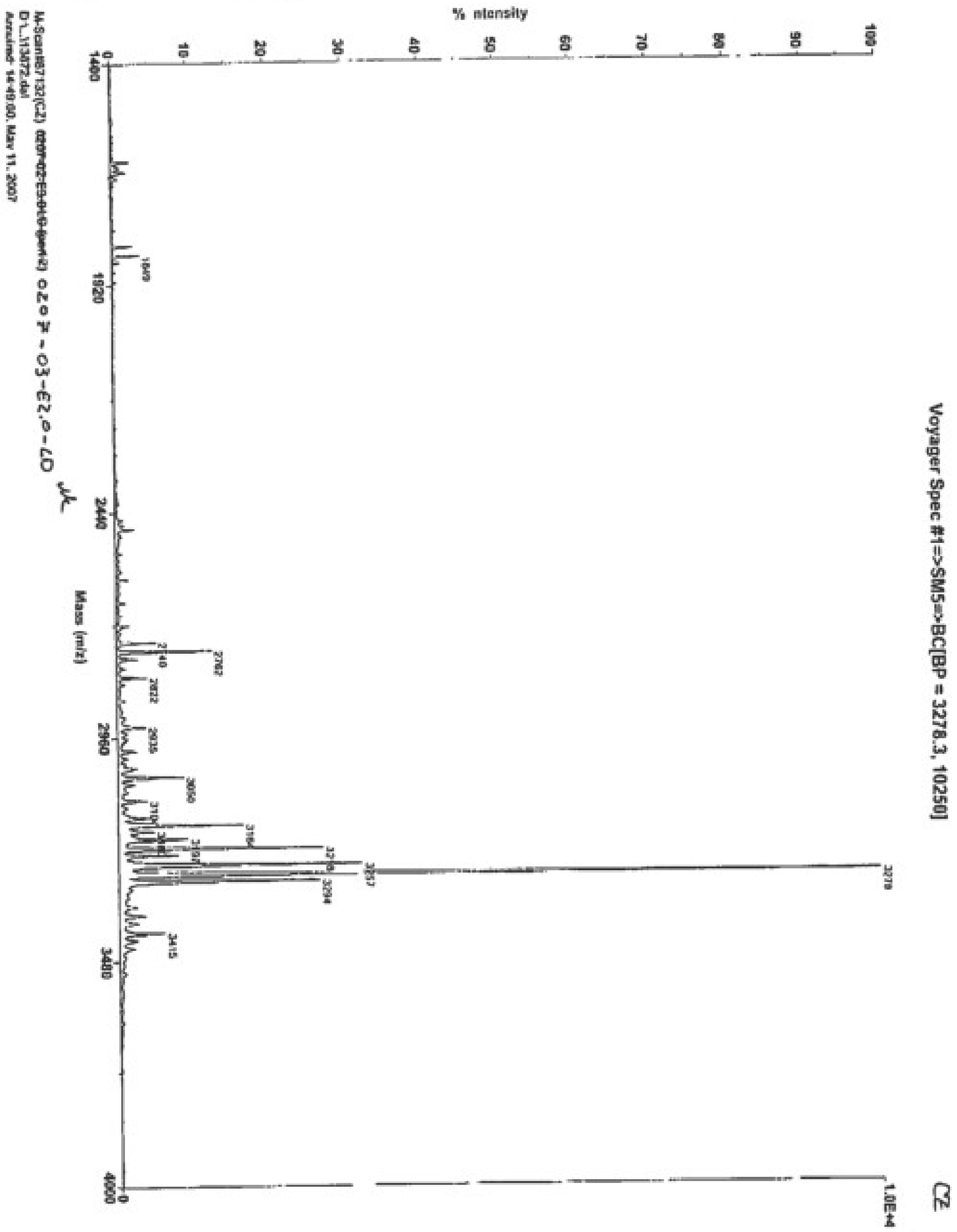

B G2-OH

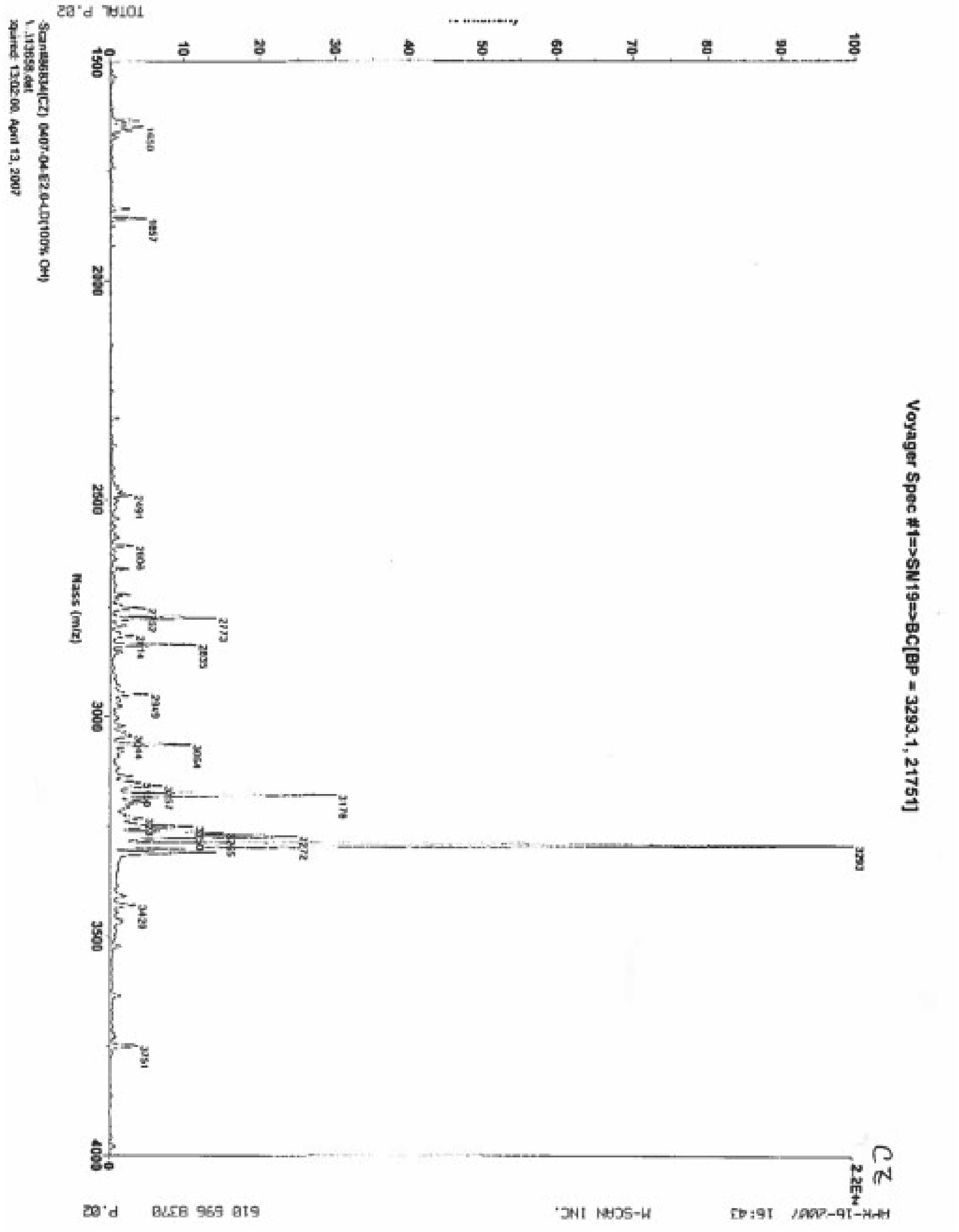
Voyager Spec #1=>SN19=>BC[BP = 3293.1, 21751]
Mass (m/z)
3293
3176
2773
2835
3054
2.2E+4

C G2-SA

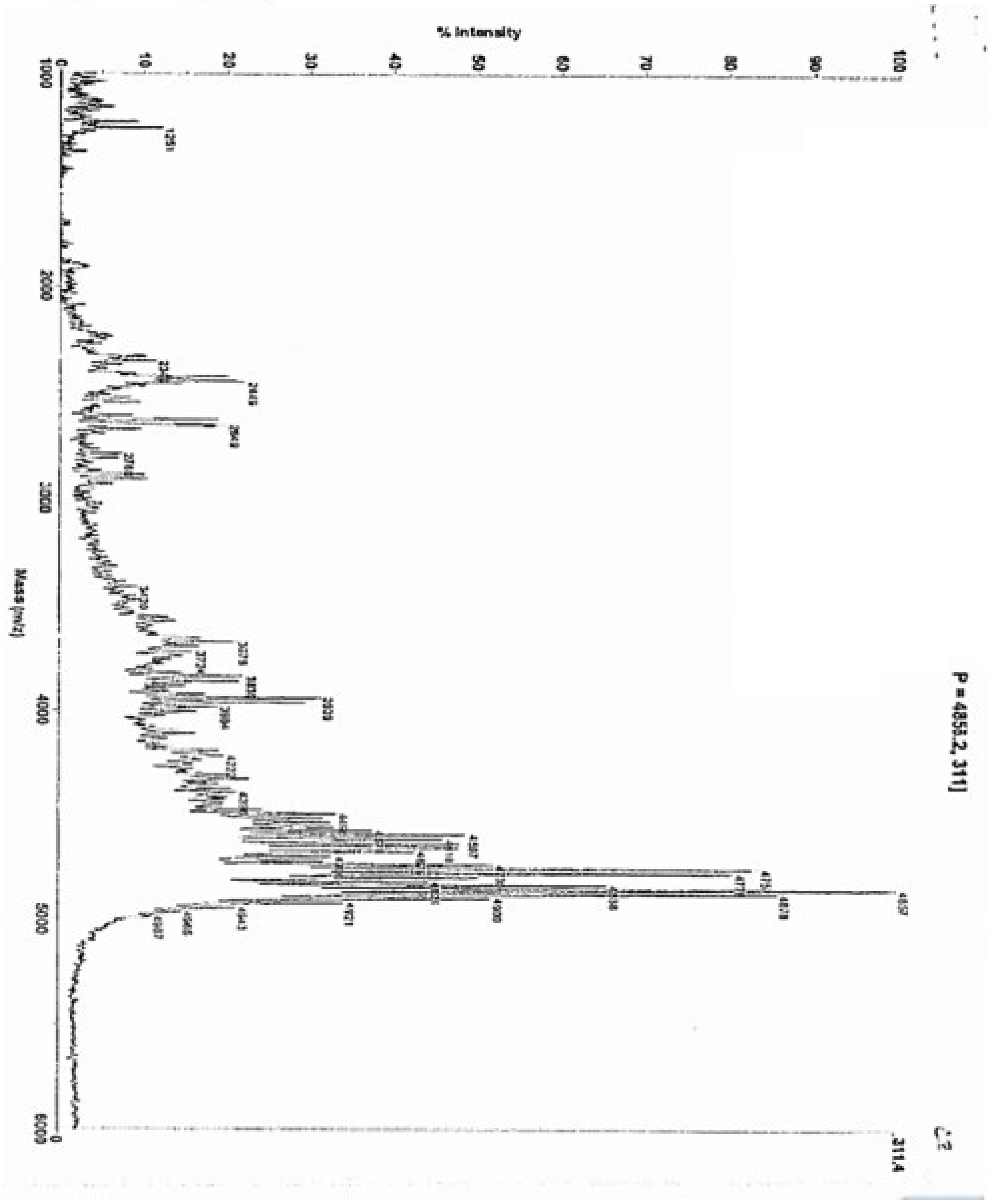

D G2-COONa

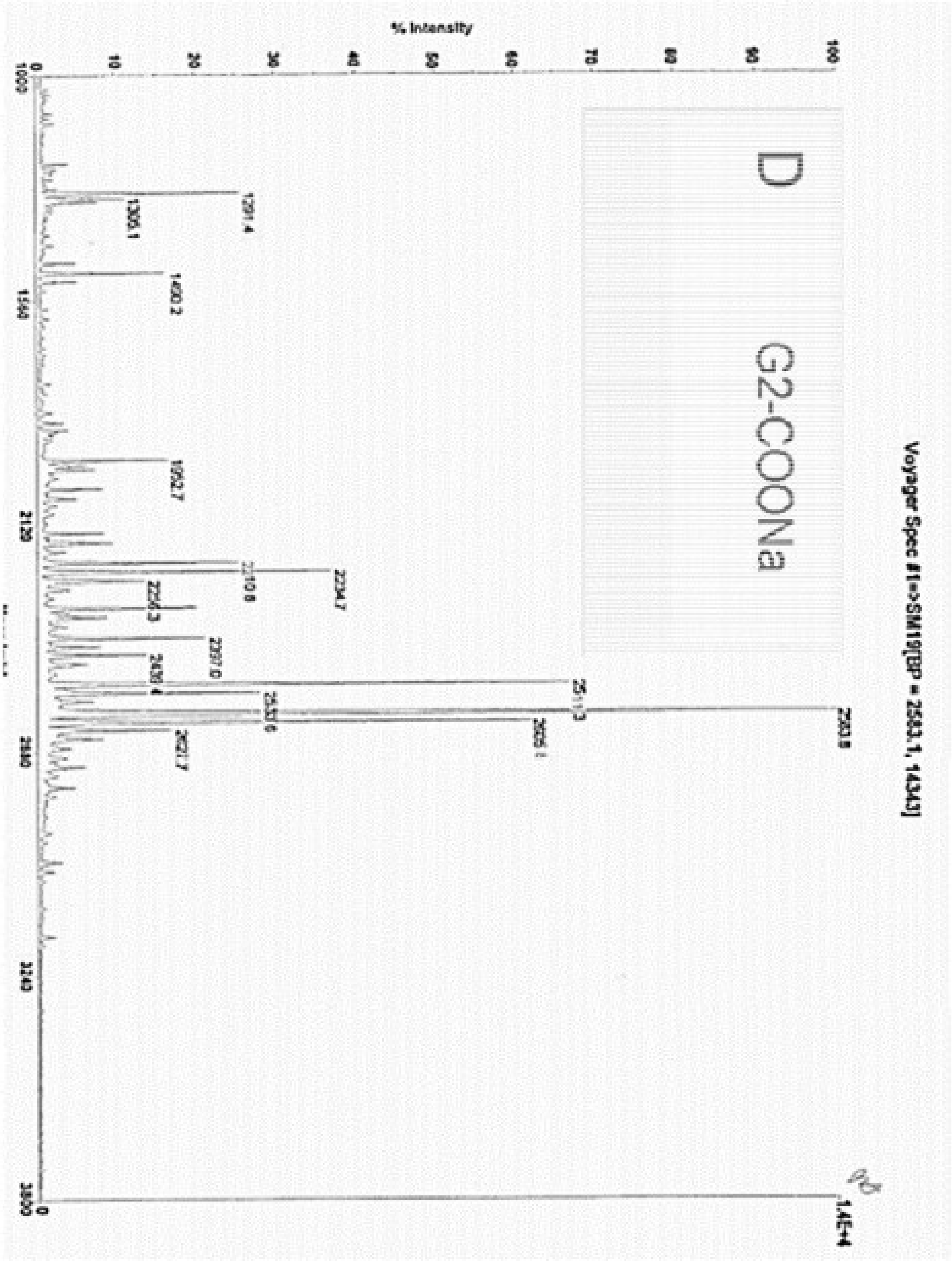

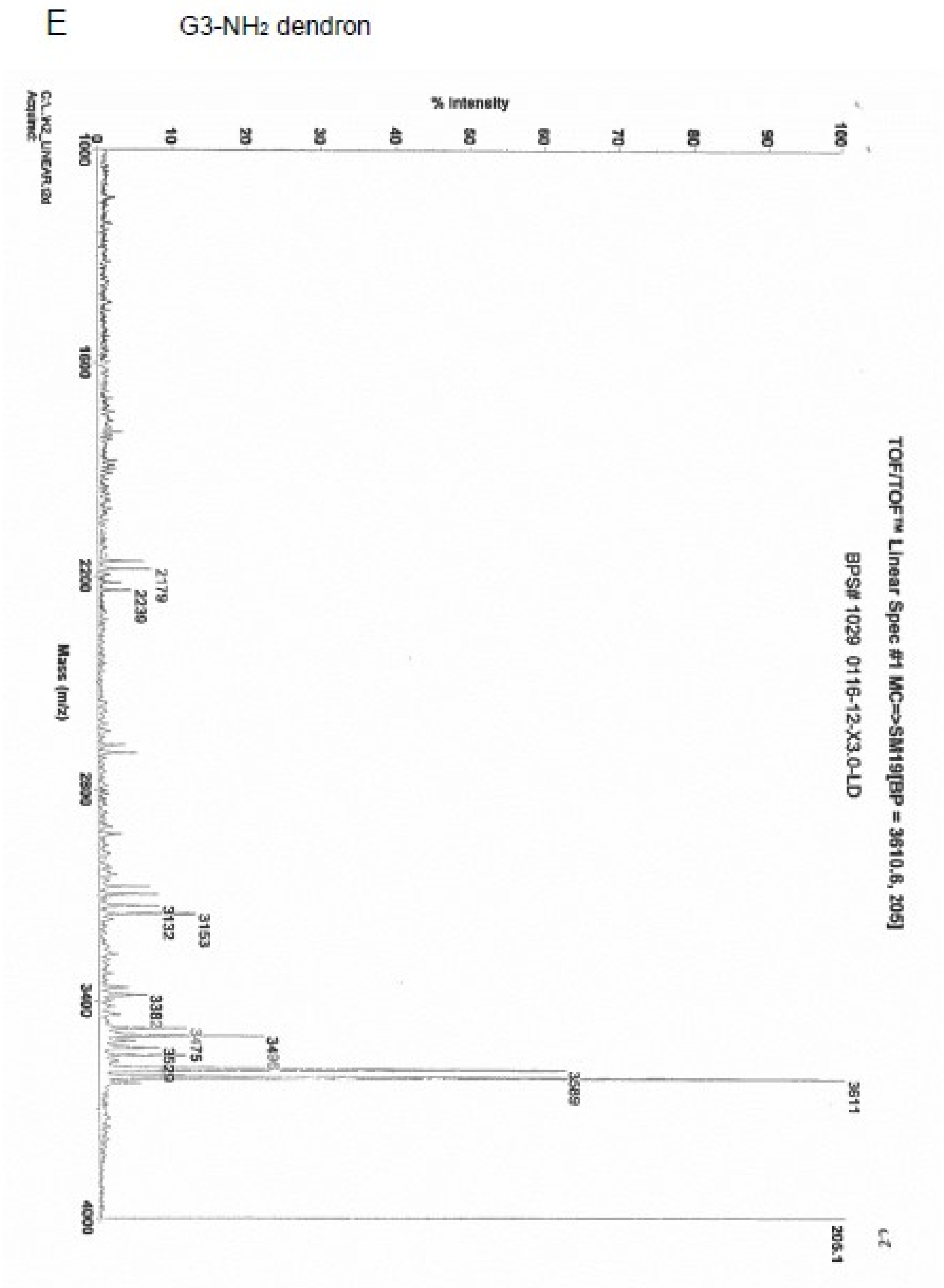

Figure S5. MALDI-TOF mass spectra of G2-NH_2 (**A**); G2-OH (**B**); G2-SA (**C**); G2-COONa (**D**) dendrimers and G3-NH_2 dendron (**E**). The data were provided by Dendritech, Inc (Midland, MI USA).

A G2-NH_2

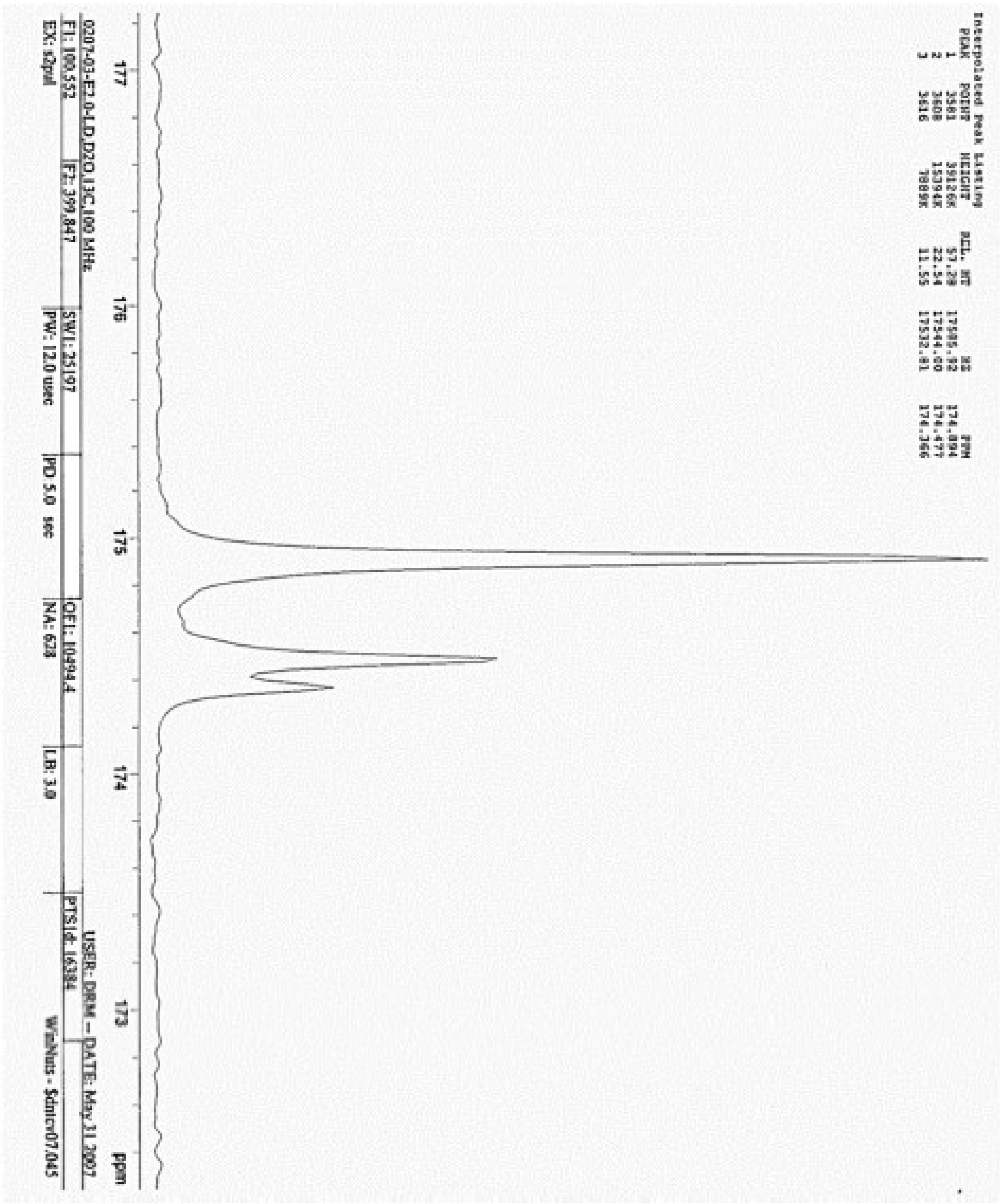

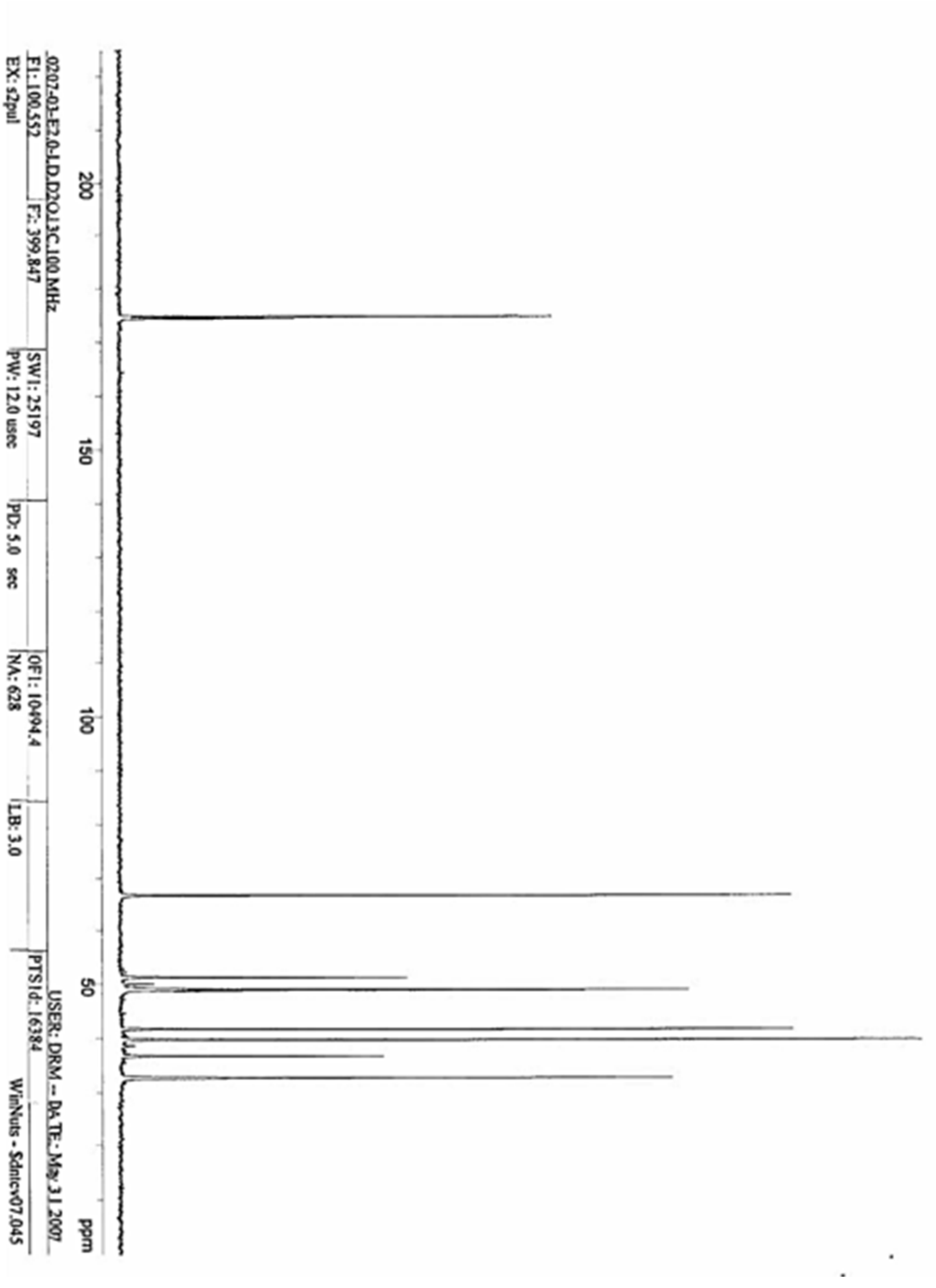
F1: 100.552
F2: 399.847
SW1: 25197
PW: 12.0 usec
PD: 5.0 sec
OF1: 10494.4
NA: 628
LB: 3.0
PTSId: 16384
USER: DRM -- DATE: May 31 2007
200
150
100
50
ppm

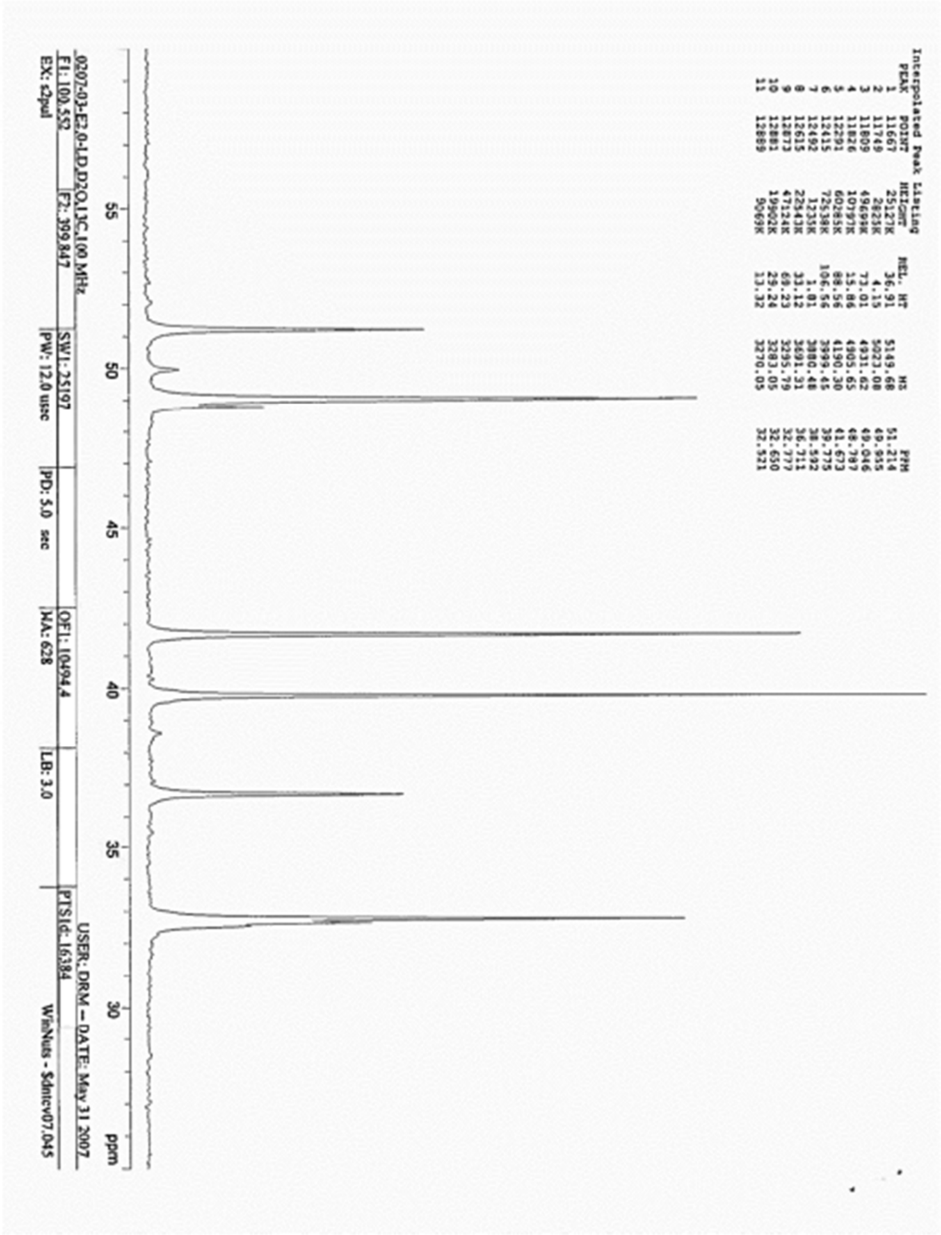

B G2-OH

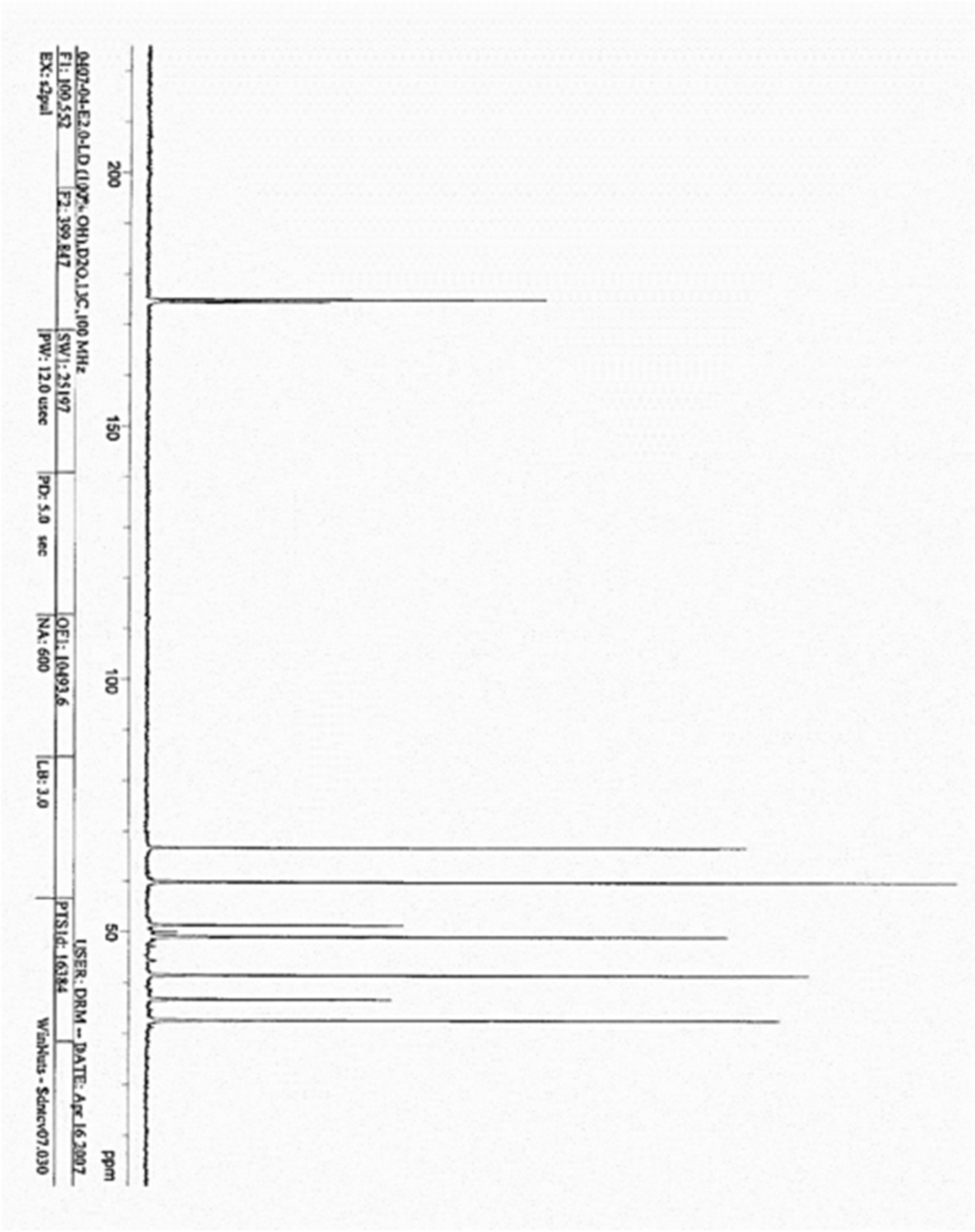

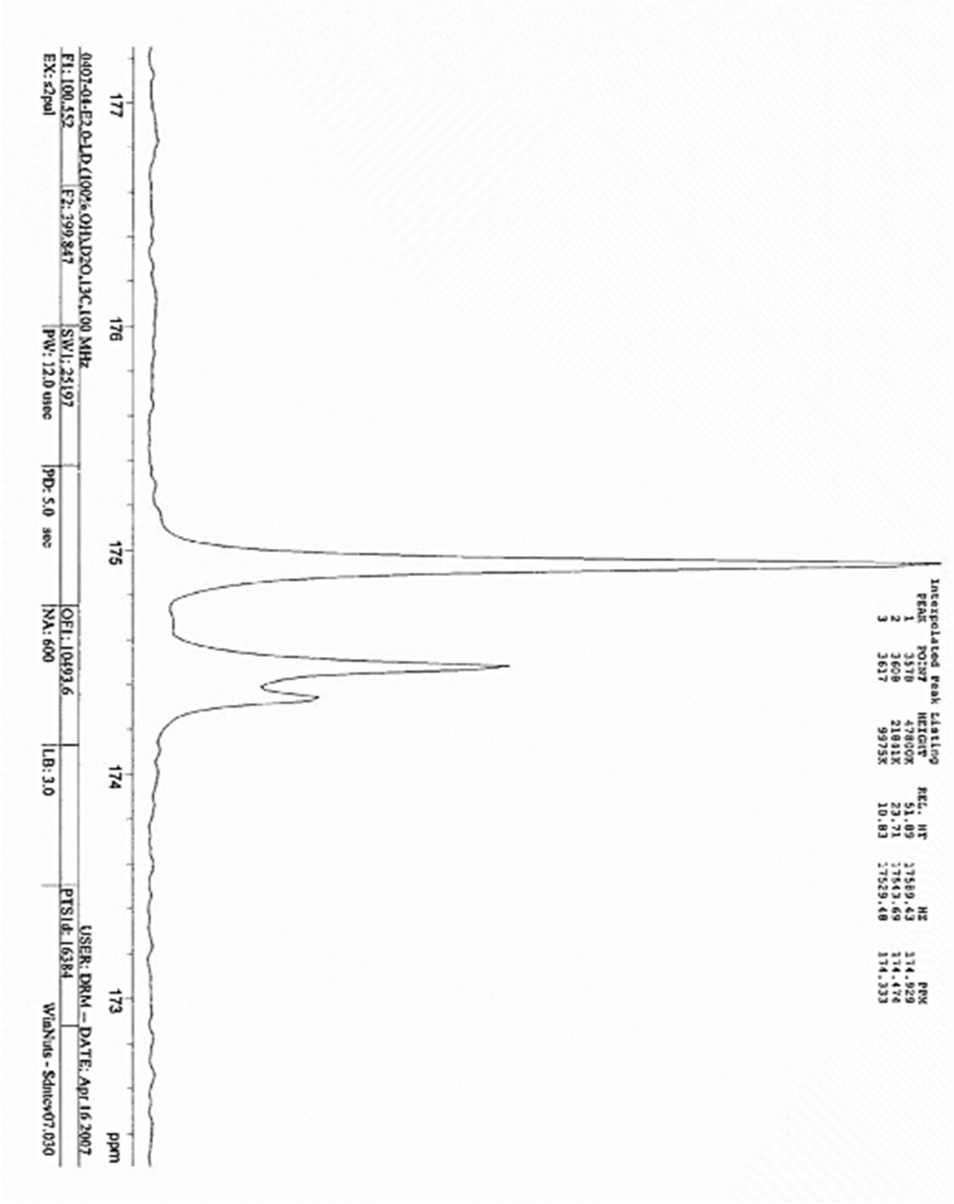
177
176
175
174
173
ppm
0401-04-E2.0-LD.(100%-OH)D2O,13C,100 MHz
F1: 100.452
F2: 399.847
SW1: 25197
OF1: 10493.6
PTS1d: 16384
USER: DRM -- DATE: Apr 16 2007
EX: s2pul
PW: 12.0 usec
PD: 5.0 sec
NA: 600
LB: 3.0
WinNuts - Sdntcv07.030
Interpolated Peak Listing

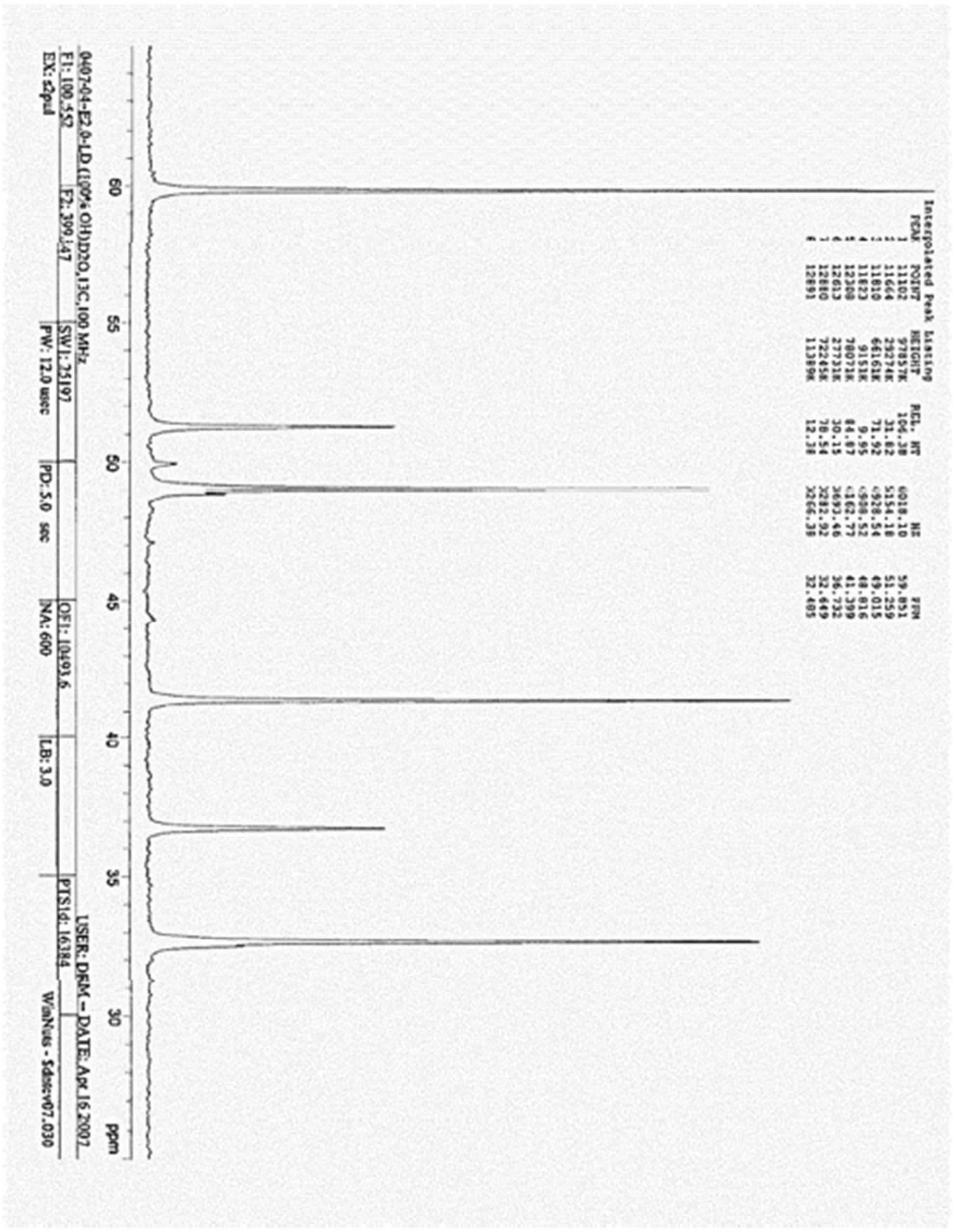

C G2-SA

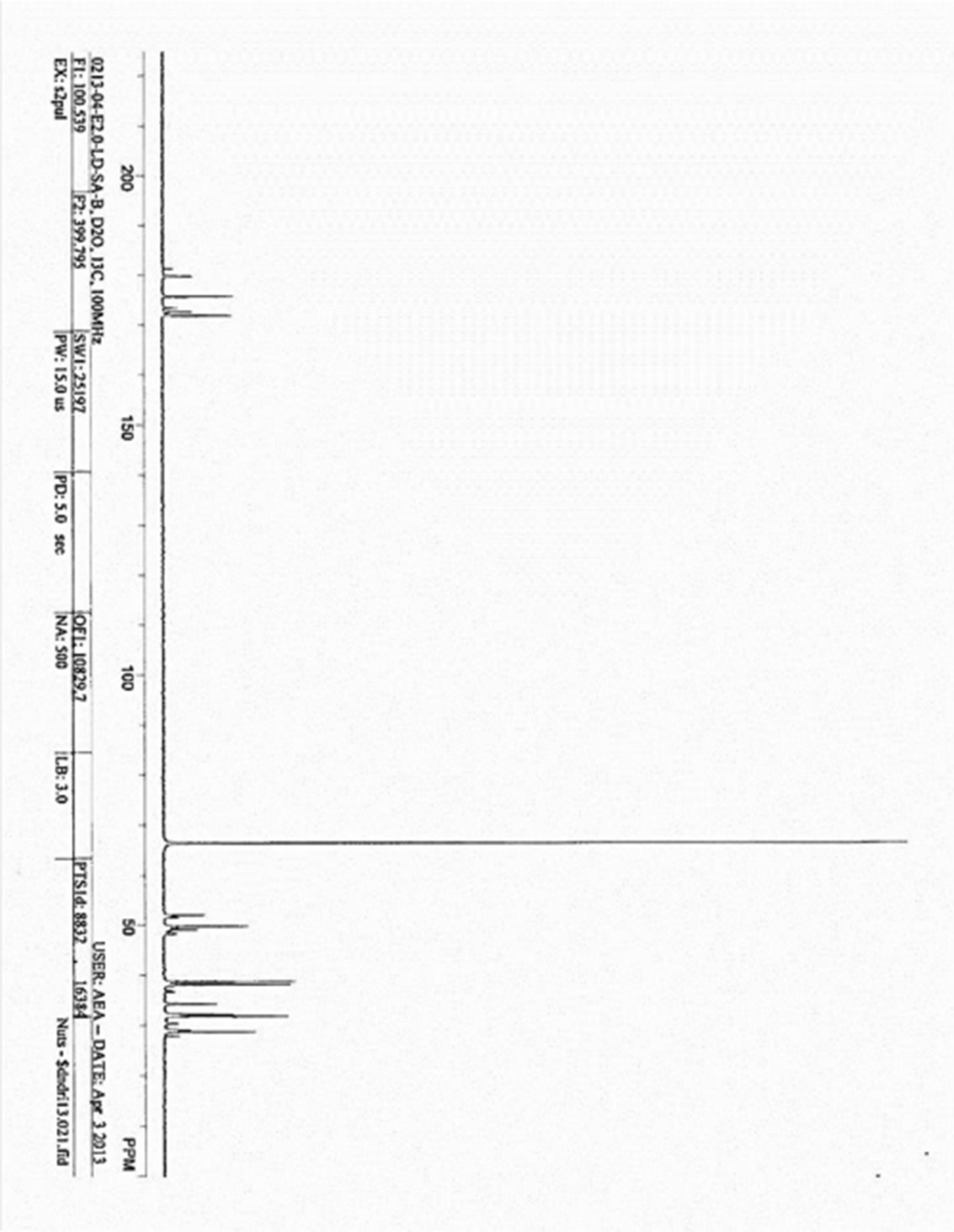
0213-04-E2.0-LD-SA-B, D2O, 13C, 100MHz
F1: 100.539
F2: 399.795
SW1: 25192
PW: 15.0 us
PD: 5.0 sec
OF1: 10829.7
NA: 500
LB: 3.0
PTS1d: 8832 . 16384
EX: s2pul
USER: AEA – DATE: Apr 3 2013
Nuts - $dodri13021.fid
200
150
100
50
PPM

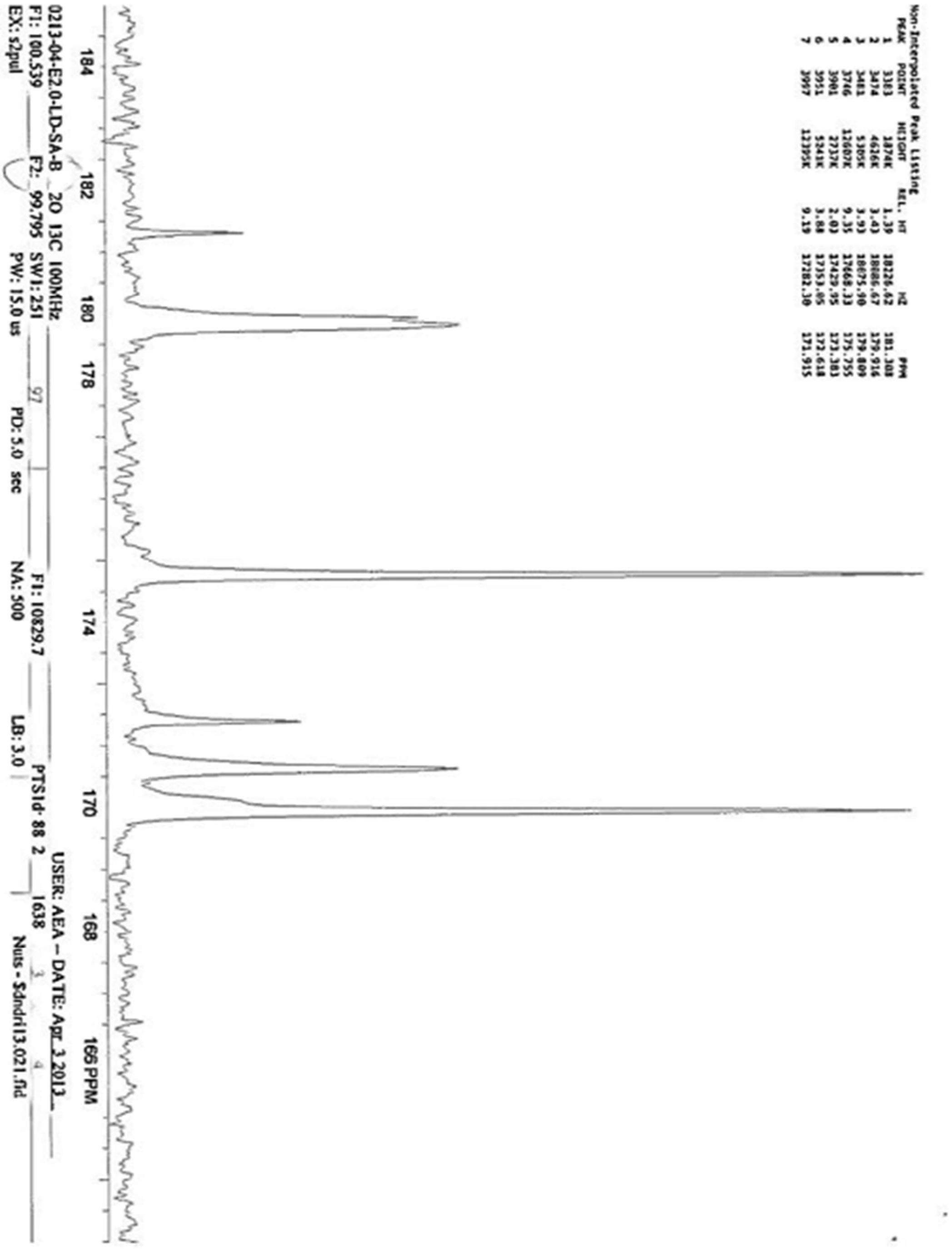
Non-Interpolated Peak Listing
0213-04-E2.0-LD-SA-B 2O 13C 100MHz
USER: AEA – DATE: Apr 3 2013
F1: 100.539 F2: 99.795 SW1: 251 FI: 10829.7 PTS1d: 88 2 1638
EX: s2pul PW: 15.0 us PD: 5.0 sec NA: 500 LB: 3.0 Nuts - $dndri13.021.fid
184
182
180
178
174
170
168
166 PPM

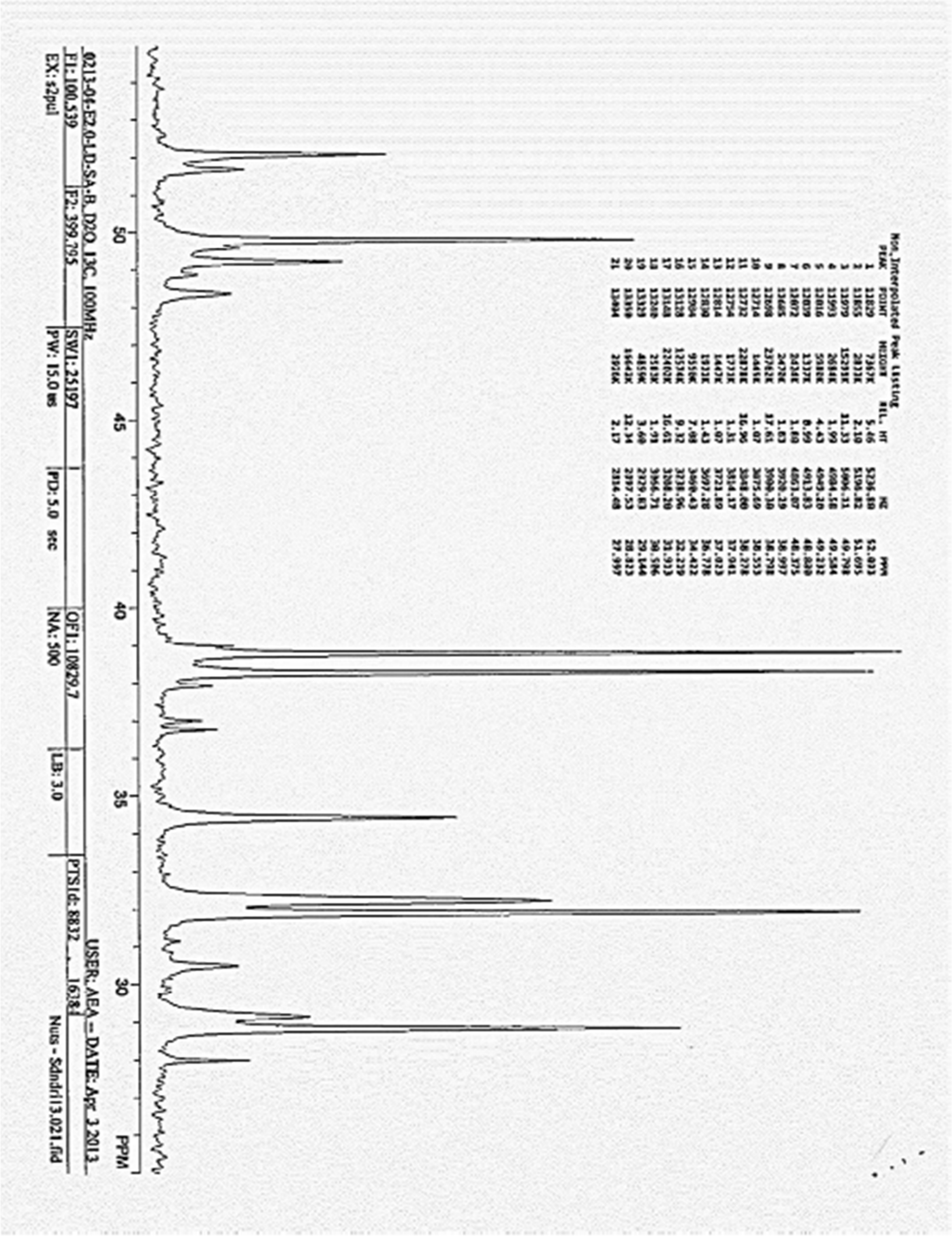
50
45
40
35
30
PPM

D G2-COONa

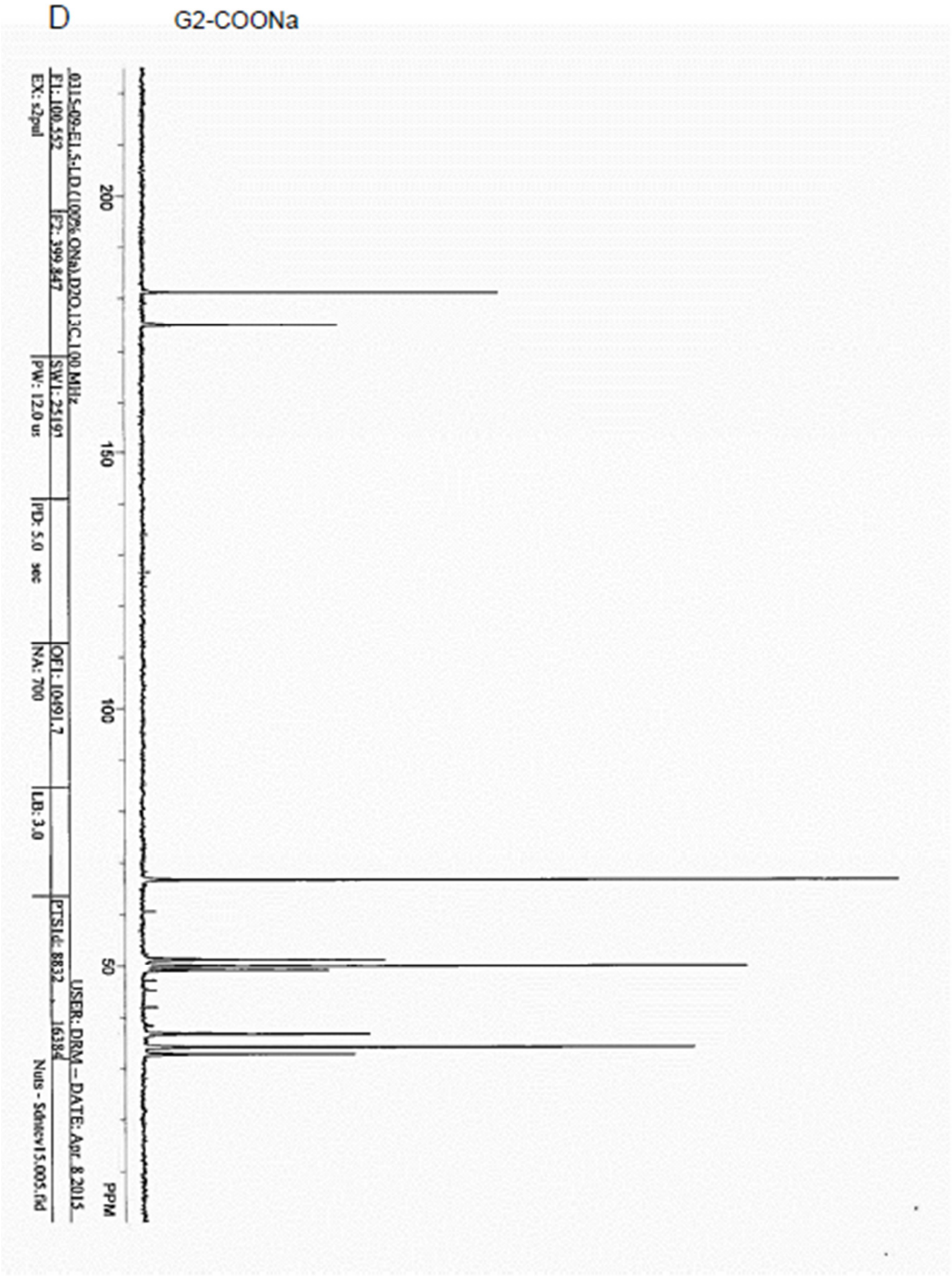

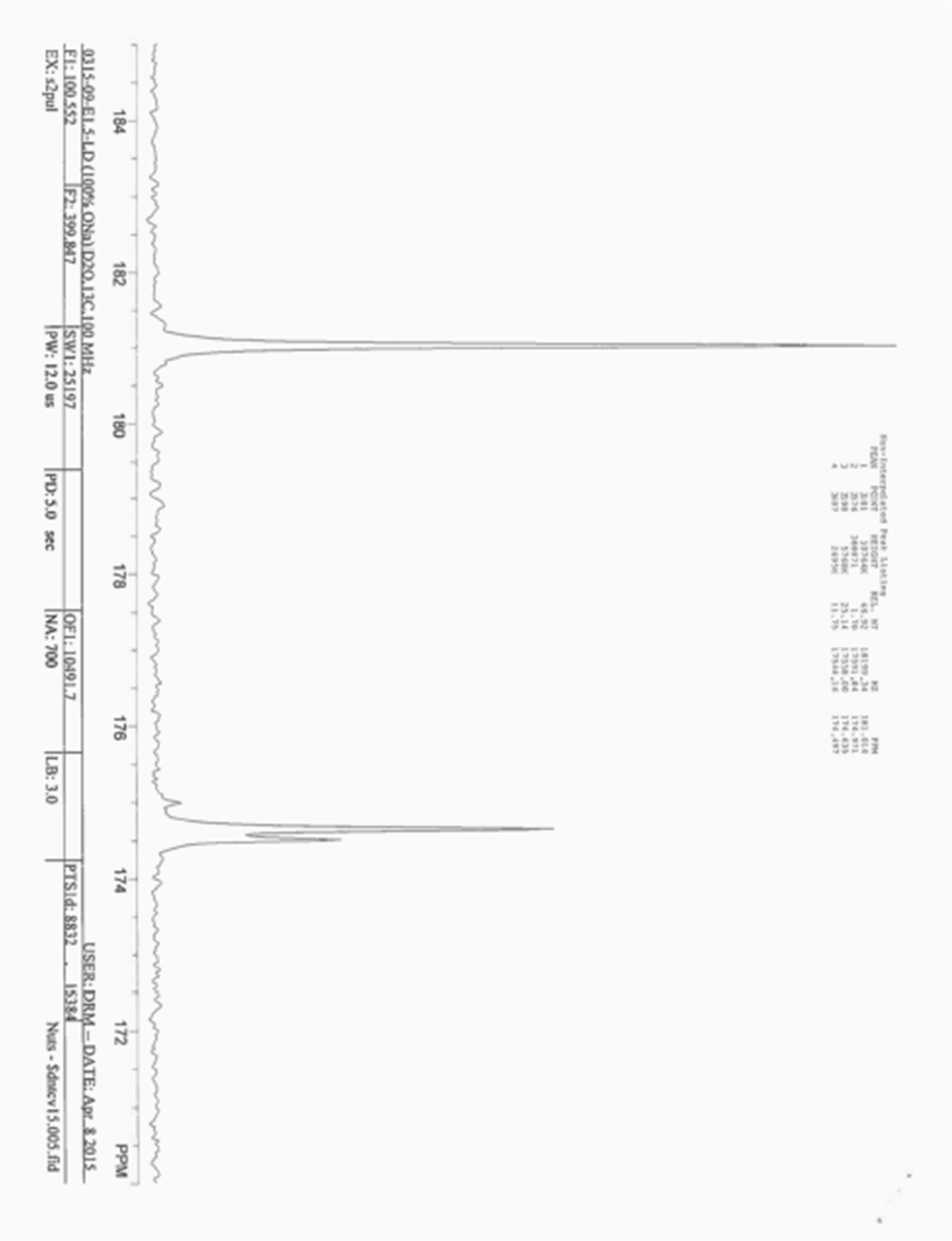
0315-09-EL5-LD (100% ONa) D2O 13C 100 MHz
F1: 100.552
F2: 399.847
SW1: 25197
EX: s2pul
PW: 12.0 us
PD: 5.0 sec
NA: 700
OF1: 10491.7
LB: 3.0
PTS1d: 8832
15384
USER: DRM -- DATE: Apr. 8 2015
Nuts - Sdmcv15.005.fid
184
182
180
178
176
174
172
PPM

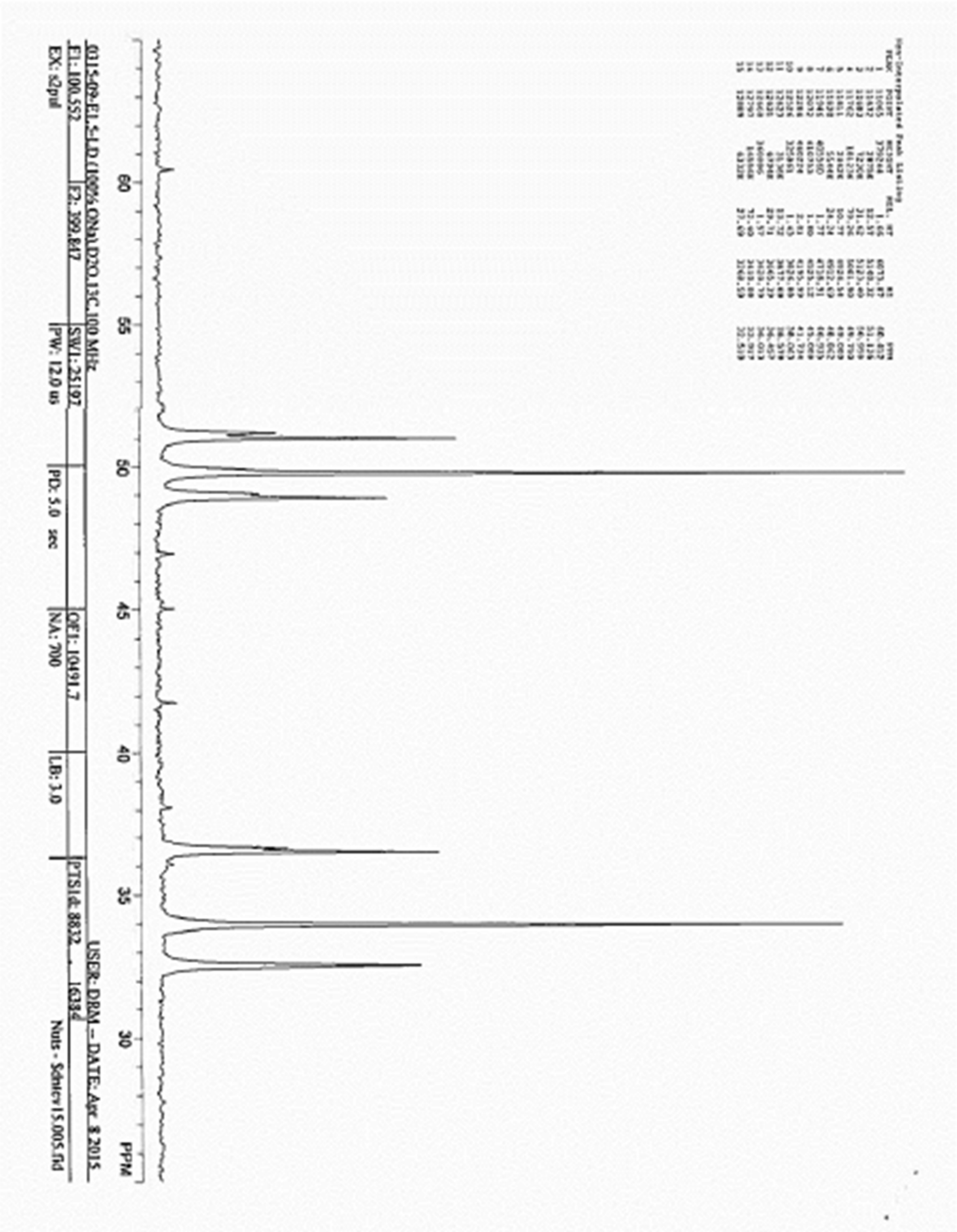
EX: s2pul
PW: 12.0 us
PD: 5.0 sec
NA: 700
LB: 3.0
SW1: 25197
OF1: 10491.7
PTS1d: 8832 . 16384
USER: DRM -- DATE: Apr 8 2015
Nuts - Sdniev15.005.fid
60
55
50
45
40
35
30
PPM

E G3-NH_2 dendron

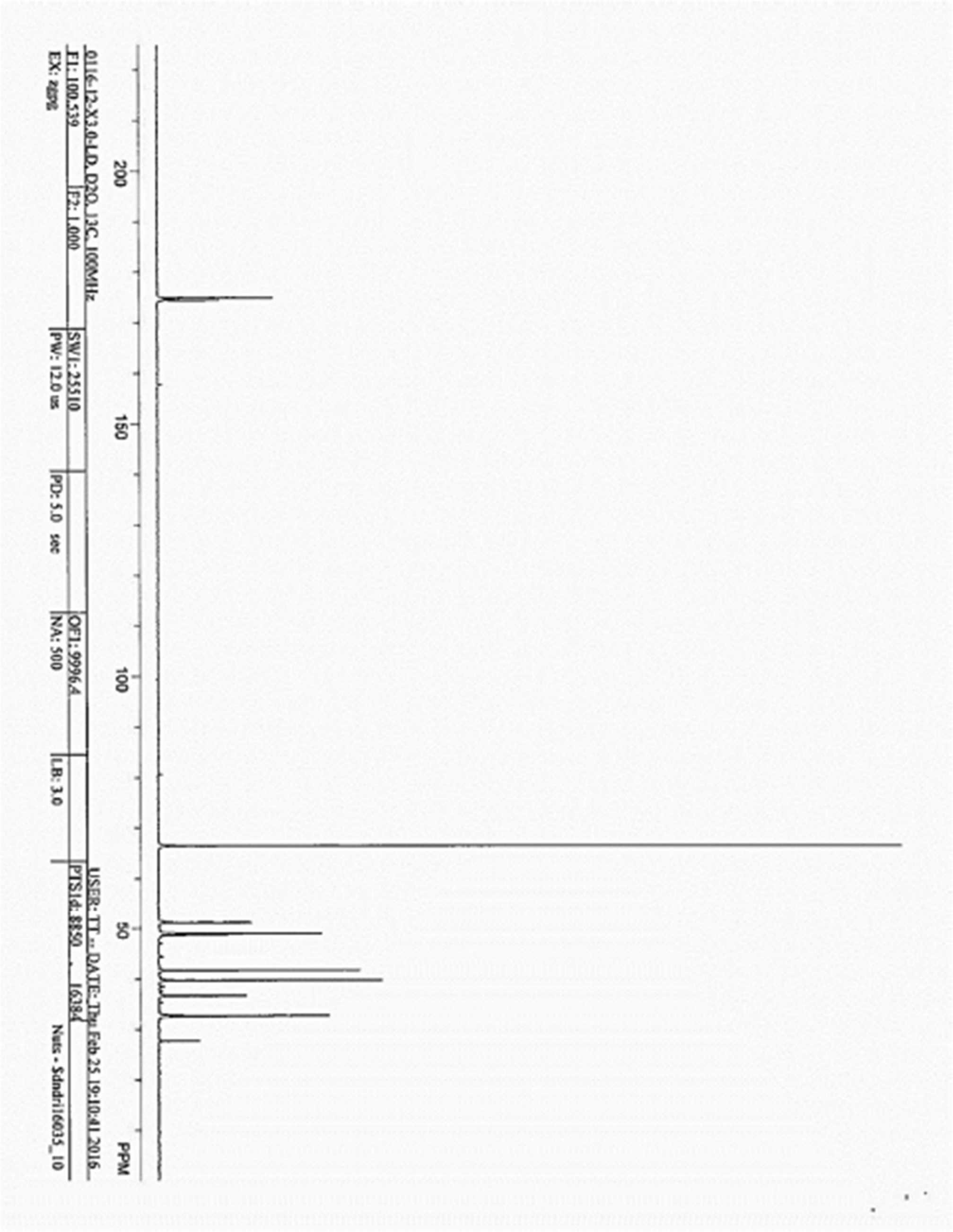

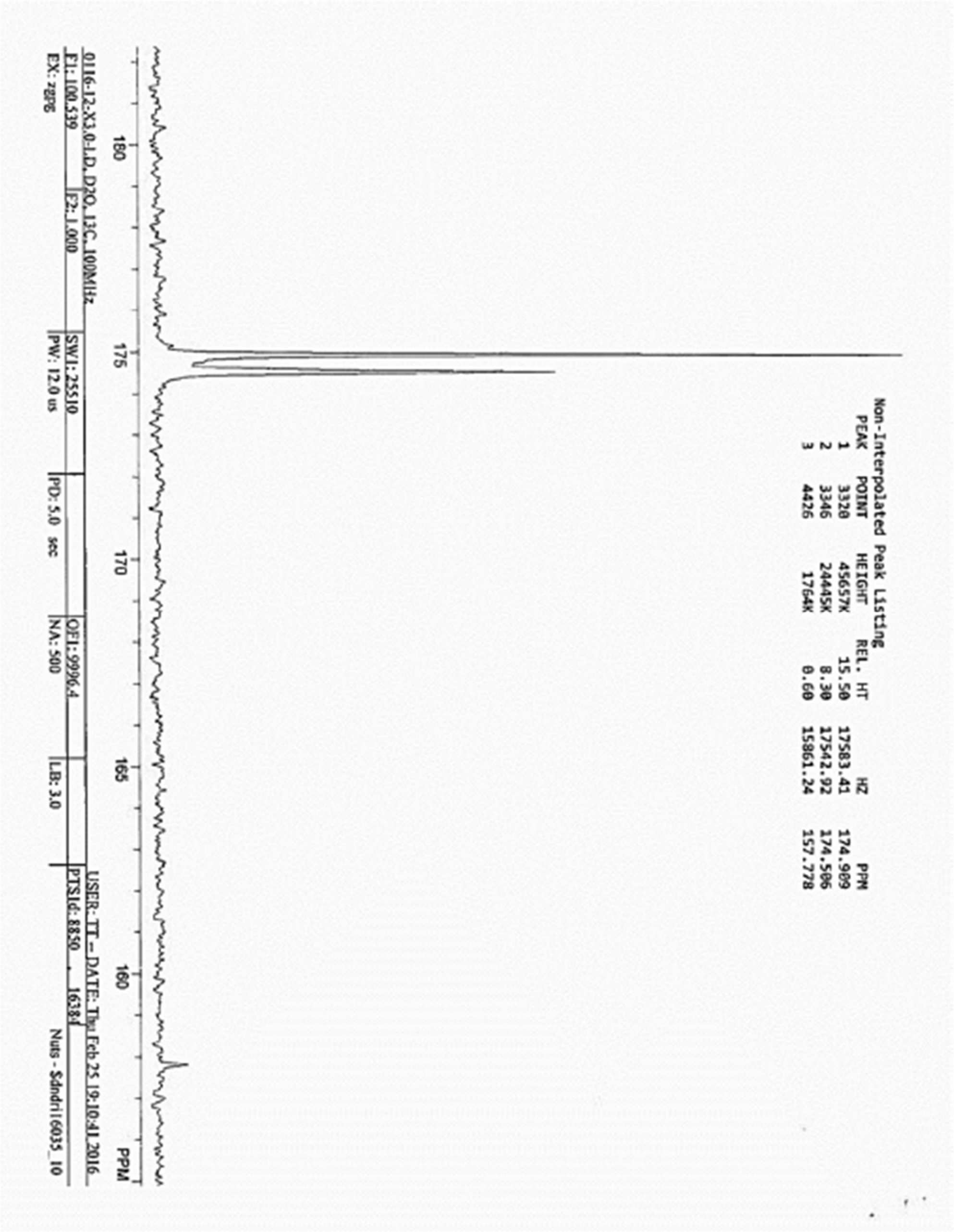
Non-Interpolated Peak Listing
PEAK POINT HEIGHT REL. HT HZ PPM
1 3320 45657K 15.50 17583.41 174.909
2 3346 24445K 8.30 17542.92 174.586
3 4426 1764K 0.60 15861.24 157.778
180
175
170
165
160
PPM
F1: 100.539
F2: 1.000
EX: zgpg
SW1: 25510
PW: 12.0 us
PD: 5.0 sec
OF1: 9996.4
NA: 500
LB: 3.0
PTS1d: 8850

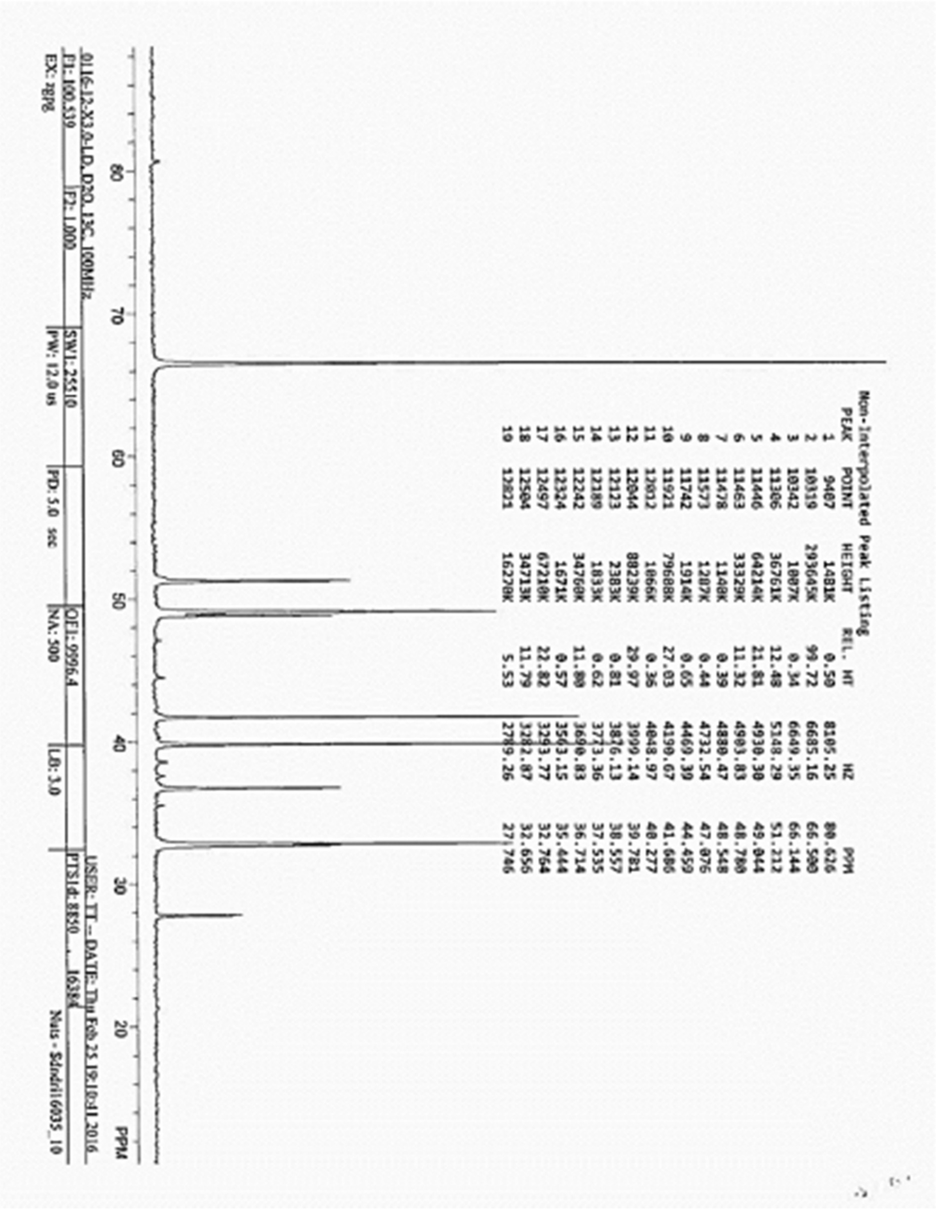

Figure S6. Characterization of generation 2 PAMAM dendrimers and generation 3 PAMAM-NH_2 dendron by 13 C NMR. The data were provided by Dendritech, Inc.(Midland, MI USA), (**A**) 13 C NMR of G2-NH_2. (**B**) 13 C NMR of G2-OH; (**C**) 13 C NMR of G2-SA; (**D**) 13 C NMR of G2-COONa. (**E**) 13 C NMR G3-NH_2 dendron.
